# A multi-level analysis of motor and behavioural dynamics in 9-month-old preterm and term-born infants during changing emotional and interactive contexts

**DOI:** 10.1038/s41598-024-83194-w

**Published:** 2025-01-06

**Authors:** Yu Wei Chua, Lorena Jiménez-Sánchez, Victoria Ledsham, Sinéad O’Carroll, Ralf F. A. Cox, Ivan Andonovic, Christos Tachtatzis, James P. Boardman, Sue Fletcher-Watson, Philip Rowe, Jonathan Delafield-Butt

**Affiliations:** 1 Strathclyde Institute of Education, University of Strathclyde, Lord Hope Building, Glasgow, G4 0LT UK; 2 Laboratory for Innovation in Autism, University of Strathclyde, Graham Hills Building, Glasgow, G1 1QE UK; 3 Centre for Reproductive Health, Institute for Regeneration and Repair, University of Edinburgh, 4-5 Little France Drive, Edinburgh BioQuarter, Edinburgh, EH16 4UU UK; 4 Translational Neuroscience PhD Programme, Salvesen Mindroom Research Centre, The University of Edinburgh, Kennedy Tower, Morningside Terrace, Edinburgh, EH10 5HF UK; 5 Department of Psychology, University of Groningen, Grote Kruisstraat 2/1, 9712 TS Groningen, The Netherlands; 6 Department of Electronic and Electrical Engineering, University of Strathclyde, Glasgow, UK; 7 Salvesen Mindroom Research Centre, The University of Edinburgh, Kennedy Tower, Edinburgh, EH10 5HF UK; 8 Department of Biomedical Engineering, University of Strathclyde, Glasgow, UK; 9 Department of Public Health, Policy and Systems, Institute of Population Health, University of Liverpool, Liverpool, L69 3GF UK

**Keywords:** Multiscale entropy, Permutation entropy, Recurrence quantification analysis, Preterm birth, Sensors, Behavioural micro-analysis, Emotion, Sensorimotor processing, Computational biology and bioinformatics, Human behaviour

## Abstract

Computational analysis of infant movement has significant potential to reveal markers of developmental health. We report two studies employing dynamic analyses of motor kinematics and motor behaviours, which characterise movement at two levels, in 9-month-old infants. We investigate the effect of preterm birth (< 33 weeks of gestation) and the effect of changing emotional and social-interactive contexts in the still-face paradigm. First, multiscale permutation entropy was employed to analyse acceleration kinematic timeseries data collected from Inertial Measurement Unit (IMU) sensors on infants’ torso, wrists, and ankles (N = 32: 10 term; 22 preterm). Second, Recurrence Quantification Analysis was used to characterise patterns of second-to-second behavioural changes, from observationally coded behavioural timeseries on infants’ emotional self-regulation (N = 111: 61 term; 50 preterm). We found frequency-specific effects of context on permutation entropy. Relative to infants born at term (> 37 weeks of gestation), infants born preterm showed greater permutation entropy in their left ankle and torso movements, but not in right ankle or wrist movements. We did not find effects of preterm birth or emotional context on micro-level behavioural dynamics. Our methodology and findings inform future work using multiscale entropy to study infant development. Dynamic analysis of behaviour is a relatively young field, and applications to emotional self-regulation requires further methodological development.

**Supplementary Information:**

The online version contains supplementary material available at 10.1038/s41598-024-83194-w.

## Introduction

Preterm birth is defined as birth before 37 weeks of gestation^1^. Children born preterm are at risk of difficulties with motor coordination and socioemotional functioning^2^. Motor developmental delays of infants born preterm have commonly been studied using psychological assessment scales. Socioemotional behaviours in infants born preterm are typically assessed using psychological scales, or quantified in experimental situations at an aggregate level. However, these methods discard rich information on moment-to-moment changes in how movement and behaviours unfold over time. This study advances the dynamic analysis of movement and behaviour, applying it to the effects of preterm birth and contextual changes in the still-face paradigm, an established developmental paradigm.

Analyses of dynamics can be achieved through a range of methods^3^. For example, spectral analysis uses Fourier transformation to decompose the timeseries into sine-wave components of different frequencies. Recurrence quantification analysis describes the state of the system state relative to its behaviour in the past^4^. It involves a graphical visualisation of the times when the system revisits a previous state. The dynamics of the system can then be described, including in terms of percentage of recurrences, as well as the pattern of recurrences. By reconstructing the phase space of a timeseries using Takens Theorem^5^, one can also plot the trajectory of the timeseries from one state to another in dimensional space. Entropy methods characterise the unpredictability or irregularity of the timeseries by quantifying the repetition of temporal patterns in the timeseries^6^, which indicates that the timeseries is evolving in the same way as it has occurred before. Each method may characterise a different aspect of the timeseries, such as its stability, irregularity, or state transitions, with its own strengths and limitations.

Dynamic analysis of motor kinematics in infancy have demonstrated promise in studying development, particularly in using measures of entropy. In movement science, a higher or more irregular movement entropy is used to indicate greater movement complexity, which relates to the ability of using different movement strategies (e.g. characterised by variation in kinematic patterns) to accomplish a specific task^7–9^. The term complexity has therefore been used interchangeably with entropy. Movement complexity changes over motor development, generally increasing from childhood to adulthood^9^. In contrast, movement complexity shows nonlinear developmental changes in infancy. This has been described predominantly in work on the development of sitting ability^10–12^. Infants may initially develop a range of strategies leading to increased kinematic complexity, but the acquisition of sitting ability is associated with a decrease in postural movement complexity attributed to the selection of specific strategies that lead to success in postural control^13,14^.

Lower entropy in physiological and motor kinematic signals is associated with aging, disease and health risks^15,16^. Extending this to development, lower complexity of micro-level dynamics may indicate developmental risk. In line with this, lower movement complexity has been demonstrated in infants with neural tube defects^17^, in a heterogeneous group of infants experiencing early life risks including preterm birth and medical complications^18^, and in infants with high familial likelihood for autism^19^, compared to infants who did not experience these risks. Several of these studies focus on changes in motor complexity relative to the onset of a specific motor skill, and children experiencing developmental risks tend to be assessed at a later age as a result (as large as a 10 month difference in test age)^11,20^. Further, only three studies have examined developmental differences in motor complexity in infants born preterm (< 37 or < 33 weeks of gestation)^10,12,21^, of which only one study included a comparison group of infants born at term age^10^. The impact of developmental risk on lower motor complexity is also likely to be an oversimplistic hypothesis. Researchers have theorised how motor complexity may be an important for the development of skilled movement—where too much or too little variability may hinder learning of skilled patterns of movement, and too little may affect the ability to adapt motor patterns to environmental perturbations.

Effects of context on movement complexity have also been identified^22–26^. For example, visual feedback increases movement complexity and decreases motor error^27^. Cognitive and attentional resources allocated to motor coordination also affect movement complexity^25,26^. Evidence is more limited in infants, but postural movements in infants showed smaller fluctuations in the presence versus absence of a toy^10,12,28^ – indicating that it is likely that motor complexity is dependent on context, such as the nature of the motor task, and the amount of cognitive and attentional resources for motor coordination.

Dynamic analysis of behaviour, which quantifies the fluctuations in behaviour over time, is an emerging field. Dynamic analysis of behaviour, also termed behavioural micro-analysis, focuses on moment-to-moment changes in behaviour rather than quantities of behaviour aggregated over time. It has proven to be useful in characterising and understanding the temporal aspects of behaviour^29^, such as in its application to the contingency of caregiver and infant behaviour in time^29^, and to characterising sequences of caregivers’ communicative behaviours^30,31^.

Here, we demonstrate as a proof of concept, the application of dynamic analysis methods on two levels of infant movement in an established developmental paradigm, the still-face paradigm. The still-face paradigm involves contextual changes between social interactive phases between the infant and caregiver, and stressful non-interactive (“still-face”) phase, where the caregiver puts on a neutral expression and stops interacting. Including a second still-face phase is more stressful for the infant^32^. Behaviour is sensitive to the socioemotional context in this paradigm^33^, with greater expressions of negative affect and self-regulatory behaviours in the stressful still-face phase. However, little is known whether the motor kinematic form of these behaviours may also contain signatures related to contextual constraints. In this paradigm, motor activity is typically analysed in terms of its overall or mean level but motor activity contains micro-patterns that may provide rich information on the underlying complex motor system producing it.

Therefore, in Study 1, we focus on entropy of motor kinematics. Previous work investigating infant movement entropy in developmentally at-risk infants have only done so at a single timescale, the sampling rate of the movement sensing device. As complex systems contain interactions at more than one level and timescale, multiscale entropy has been developed to capture complexity in biological signals better^34,35^. We advance the application of multiscale entropy in Study 1, which aims to explore the effect of preterm birth and contextual changes in the still-face paradigm on movement complexity. We hypothesise that there will be an effect of preterm birth on movement complexity as previous work have identified differences reflecting immature postural control and postural development^10^ and that this may be timescale dependent. With no prior work on movement kinematics specific to the contextual changes in the still-face paradigm, we do not make specific hypotheses regarding contextual changes.

Mean levels of behaviour have been extensively described in the still-face paradigm, with recent advances in quantifying temporal information^36–38^. In Study 2, we demonstrate an approach to characterise the micro-level dynamics of infant emotional self-regulatory behaviours in the still-face phases, in addition to characterising the amount of behaviours (results reported in a separate publication) (Chua et al., in review^39^). To robustly quantify dynamics, a timeseries needs to contain enough samples, posing a challenge to obtaining behavioural data suitable for such an analysis, as behaviour unfolds over a slower timescale. Due to this constraint and because behaviour is quantified within categories, we use Recurrence Quantification Analysis (RQA) which can be applied to short timeseries and to categorical data^40^. Study 2 aims to explore the effects of preterm-birth and a repeated stressor on micro-level behavioural dynamics of emotional self-regulatory behaviours.

## Methods

### Study design

Study 1 and Study 2 comprised infants born at term (> 37 weeks of gestation) and infants born preterm (< 33 weeks of gestation) participating in their 9 month follow up (or 9-month corrected age for preterm born infants) of the Theirworld Edinburgh Birth Cohort (TEBC)^41^, conducted in Scotland, UK. Ethical approval was obtained from the National Research Ethics Service (NRES), South East Scotland Research Ethics Committee, and NHS Lothian Research and Development Committee, in accordance with the Declaration of Helsinki. Researchers received training on Good Clinical Practice. Participants were recruited at the Simpson Centre for Reproductive Health and parents provided written informed consent^41^. Infants born preterm who were transferred to the hospital ex utero for intensive care were excluded. Infants were also excluded if they experience major congenital malformation, chromosomal abnormality, congenital infection, cystic periventricular leukomalacia, haemorrhagic parenchymal infarction, and post-haemorrhagic ventricular dilatation. At 9 month follow up, some parents also provided written consent for the use of video or photos of them or their infant to be shared with the general public including on the web.

#### Still-face paradigm

In both studies, infants and a primary caregiver participated in an extended modification of the still-face paradigm, comprising five 2-min episodes of interactions in an A-B-A-B-A structure. The caregiver is responsive during A episodes, and unresponsive during B, “still-face 1 (SF1)” and “still-face 2 (SF2)” episodes. Infants sat on a highchair during the still-face paradigm and were able to move freely in a sitting position. Infants who were unable to sit unsupported were provided a cushion for support.

### Study 1

#### Participants

Study 1 comprised infants participating in their 9 month follow up between July 2019 and March 2021, and May 2022 to August 2022, when all infants in the cohort were followed up. Infants attending the 9 month follow up appointment were recruited to the subsample if a researcher trained in carrying out motor assessment was available at the visit. Infants who did not complete at least one still-face episode were excluded from analysis. Recruitment to the subsample proceeded until a minimum of 10 infants born at term and 10 infants born preterm with complete data from each sensor location, as well as meeting inclusion criteria for the analysis (see Statistical analysis), were obtained. The maximum sample size was limited by researchers’ capacity and disruptions to data collection during the COVID-19 pandemic.

For at least 1 min, or at least 10 min. In contrast, all infants in the term group were able to sit unsupported for at least 1 or 10 min. The sample included children who are predominantly of White Scottish/British backgrounds.

Table 1 shows the demographic and clinical characteristics of the sample. Thirty two caregiver-infant dyads (29 (90.6%) mother-infant, 3 (9.4%) grandmother or father-infant) formed the analysis sample. Ten were infants born at term age (range = 36^+3^ to 42^+1^ weeks), and 22 were infants born preterm (range = 22^+1^ to 32^+5^ weeks). There was a greater proportion of male infants in the sample born preterm. Infants from both groups were of similar height, but, as expected for very preterm infants, infants in the preterm group weighed less. All infants in the term group were able to sit unsupported. In the preterm group, around a third were able to sit supported for at least a minute, and around two-thirds were able to sit unsupported for at least 1 min, or at least 10 min. In contrast, all infants in the term group were able to sit unsupported for at least 1 or 10 min. The sample included children who are predominantly of White Scottish/British backgrounds.

**Table 1:** Sample characteristics – TEBC motor subsample. Mean (SD) provided for continuous measures and n(%) for categorical measures. Motor ability measured using the Vineland Adaptive Behaviour Scales motor scale, gross motor subscale and fine motor subscale.

Characteristic	Term, N = 10	Preterm, N = 22
Age or corrected age at visit (months)	8.9 (0.3)	8.8 (0.6)
Birthweight (g)	3,470.8 (370.6)	1,448.7 (489.0)
Birthweight Z-score	0.5 (0.6)	0.2 (1.3)
Gestation (weeks)	39.4 (1.8)	29.3 (2.5)
Sex		
Male	5 (50.0%)	16 (72.7%)
Female	5 (50.0%)	6 (27.3%)
Ethnicity		
Any White ethnicity	10 (100.0%)	18 (81.8%)
Asian or Mixed ethnicity	0 (0.0%)	4 (18.2%)
Maternal degree level qualification	6 (60.0%)	4 (18.2%)
SIMD^^^ quintile		
Quintile 1, 2 or 3 (more deprived)	3 (30.0%)	10 (45.5%)
Quintile 4 or 5 (less deprived)	7 (70.0%)	12 (54.5%)
Height^#^ (cm)	71.3 (1.5)	72.0 (4.3)
Weight^#^ (kg)	9.3 (1.2)	8.7 (1.4)
Sitting ability		
Sits supported for at least a minute	0 (0%)	7 (31.8%)
Sits unsupported for at least a minute	10 (100%)	15 (68.2%)
Motor raw score	26.9 (5.4)	22.3 (6.5)
Gross motor raw score	13.5 (4.2)	11.1 (4.3)
Fine motor raw score	13.4 (2.5)	11.1 (3.0)
Any comorbidity of preterm birth^+^	NA	12 (54.5%)

^^^SIMD – Scottish Index of Multiple Deprivation is a measure of neighbourhood deprivation, ranked from the most to the least deprived areas.

^#^1 infant born preterm, and 1 infnat born at term did not have anthropometry data on height and weight, not collected due to health and safety procedures during the COVID-19 pandemic.

^+^This included early or late onset sepsis, medical or surgical necrotising enterocolitis, retinopathy of prematurity, bronchopulmonary dysplasia.

#### Equipment and procedure

**Fig. 1:**
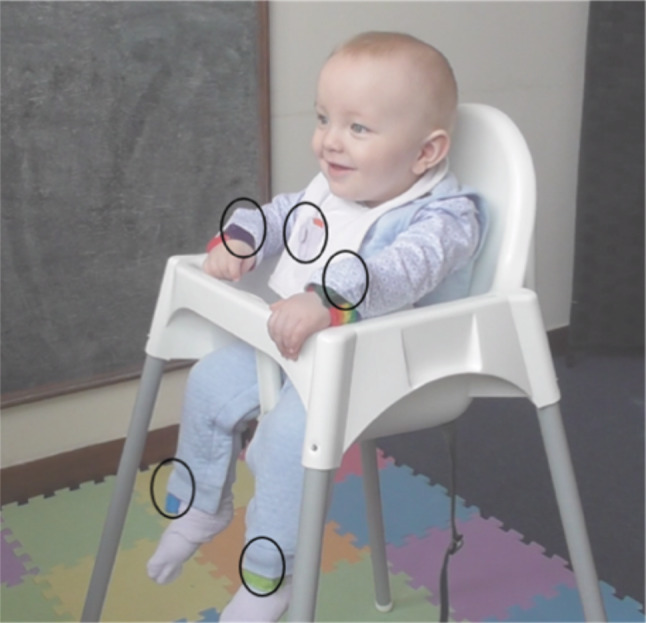
Placement of 5 IMU sensors on an infant’s torso, wrists and ankles. Black ovals mark the location of sensors hidden in clothing.

Inertial magnetic unit (IMU) sensors (Xsens MTw Awinda^42^, 100 Hz) were used to measure infant movement during the still-face paradigm. The sensors were small and lightweight (47mm x 30mm x 13mm, 16g), containing tri-axial accelerometers (± 160 ms^-2^), gyroscopes (± 2000 deg/s) and magnetometers (± 1.9 Gauss). An infant bib, two wristbands and a pair of socks with fitted pockets were used to attach sensors onto infants’ torso, wrists and ankles respectively. The bib was secured around the infants’ chest using a strap with Velcro closure around the back. Socks were put on such that sensors were located around the same level of each ankle, on the anterior side above the foot. Wristbands were adjusted so that the sensors were on the side of the wrist with the palm faced-down (i.e., wrist sensors face away from the body when the forearm is pronated). Figure 1 shows the placement of the five IMU sensors on the infants’ body. Where possible, wrist sensors were tucked under infants’ long sleeve shirts, or an additional long sleeve shirt put on to keep torso and wrist sensors out of sight. Video recordings were taken using Panasonic HC-W580 cameras (1920 × 1080 pixels, 50 frames per second). For some infants, as a technical pilot of Transistor-Transistor Logic (TTL) synchronisation of video to sensor recordings, an additional video recording was obtained using a Pi camera board (version 1.3) fitted to a Raspberry Pi (Rpi, version 3 B +) (1024 × 768 pixels, 25 fps). The MT Awinda Master station provided a wireless network enabling time synchronisation within 10μs^42^. Recordings were started and stopped using the MT manager software.

##### Sensor data processing

Sensor kinematic data on the calibrated 3D acceleration, angular velocity, magnetic field, orientation and free acceleration (i.e., acceleration after subtracting the gravity vector) was extracted using MT manager, which applies the Xsens Kalman Filter profile^42^. Sensor data was then processed in Python (version 3.7.6) on Jupyter Notebook (version 6.0.3).

##### Sensor data segmentation and filtering

Sensor data was first segmented into a timeseries for the entire still-face paradigm. This was done by identifying the video time when the sensor started recording. A low pass 20 Hz Butterworth filter (*SciPy* library) was applied to remove high frequency noise or artefactual effects on the acceleration profile. Filtering retaining 90% of the spectral power, in line with the spectral power profile of human movement in frequencies lower than 20–30 Hz. A low pass filter is suitable when the phenomena of interest obey relatively slow dynamics where high frequency is considered a nuisance. For further details on segmentation and filtering, see Supplemental material Sect. 1.1.1 and 1.1.2.

##### Acceleration magnitude

A time series of acceleration magnitude (root-mean-square acceleration) was calculated from the vector sum of the tri-axial free acceleration at each time point. Following this, data was further segmented into timeseries containing 12,000 samples, corresponding to each 2-min still-face phase (Play, SF1, R1, SF2, R2).

##### Multiscale permutation entropy

Each timeseries of acceleration magnitude, from each still-face phase, was processed to obtain permutation entropy. Multiscale permutation entropy was obtained, for 50 scale factors using the Improved Multiscale Permutation Entropy algorithm^43^, adapted for Python computing language in this study. The scale factor corresponds to timescale by multiplying by 1/sampling frequency, so the highest scale factor corresponding to a timescale of 0.5s for a sampling rate of 100 Hz). Multiscale entropy involves coarsegraining the original signal by averaging every *n* points to obtain a signal related to activity at that scale. Improved Multiscale Permutation Entropy was selected to produce more reliable estimates of permutation entropy with relatively short time series^43–45^ (due to coarsegraining, the timeseries at the highest scale factor of 50, would comprise only 240 data points). The parameters scale factor = 50, lag = 1 and m = 4 were used. The optimisation process for the selection of the embedding dimension *m* is described in the first author’s doctoral thesis (Chua, 2022, p243—245).

#### Measures

##### Demographic and clinical data

Clinical data on infants’ prematurity status, gestational age and birthweight at birth, singleton/twin status, and demographic data on infants’ sex, ethnicity and common comorbidities of preterm birth (early or late onset sepsis, medical or surgical necrotising enterocolitis, retinopathy of prematurity, bronchopulmonary dysplasia)^46^ were collected at the neonatal time point of the TEBC from electronic medical records. Neighbourhood deprivation (assessed using the Scottish Index of Multiple Deprivation (SIMD)) was collected at 9-month follow up from questionnaire-report of their current address postcode. SIMD is a multidimensional score generated by the Scottish government which ranks the deprivation levels of localities according to measures of income, employment, health, education, geographic access to services, crime and housing. Anthropometry data (height and weight) was collected by a clinical research nurse at the 9-month follow up. Sitting ability was derived from questions on sitting obtained using the Vineland Adaptive Behaviour Scales (VABS) (Comprehensive Parent Interview form) gross motor subscale. Based on caregiver’s responses to a prompt about sitting. The interviewer scores three items (sits supported for at least 1 min, sits unsupported for at least 1 min, and sits unsupported for at least 10 min) as “never”, “sometimes” or “usually”. The three items were reduced into a binary measure of sitting ability (sits unsupported or sits supported).

##### Behavioural data

Behavioural data was available for a subset of infants, recruited before March 2019. Emotional self-regulation data on a range of perceptuomotor, attentional and social behaviours was based on a derived behavioural coding scheme (see Chua et al., in review). Data on infant’s negative affective behaviours was available for a subset of infants, based on the Infant and Caregiver Engagement Phases coding scheme ^47^. Data on periods when the caregiver moves the infant was further obtained as a description of the sensor dataset, i.e., the extent it represents infants’ self-produced movement. Behavioural data was expressed as proportion of time out of each 2-min phase of the still-face paradigm.

##### Acceleration magnitude

Mean acceleration magnitude across each of the five episodes was obtained, after excluding outliers greater than 1.5 times the interquartile range to remove the influence of outliers on the mean acceleration magnitude.

##### Complexity of movement accelerations – permutation entropy

Permutation entropy was selected as a measure of movement complexity. It is robust to dynamical noise, measurement noise, and artefacts – which may be present as the infant is moving while seated on a chair. Two measures of movement complexity were considered – multiscale permutation entropy and complexity index.

##### Multiscale permutation entropy

Multiscale permutation entropy was a measure of complexity at specific timescales, and the curve structure (change in complexity across timescales) was considered. Differences in the MSE curve profile was analysed using data from SF1 only, where infant movement was independent from caregiver movement. Here we considered that human movement is the final common output of brain function and analysed the MSE curve profile based on the gamma (30-45Hz), beta (14-30Hz), alpha (8–13.5Hz), theta (4.5–7.5Hz), delta (0.5-4Hz) frequency bands, which is thought to capture different domains of neural processing. Data from 50 scale factors were reduced to the 6 scale factors corresponding to the upper and lower frequencies of 5 frequency bands. See Supplemental Material Sect. 1.1.3 and Fig. S1 and S2 for further details on the data reduction strategy.

##### Complexity index

Complexity index was a measure of overall structural complexity, estimated as the sum of permutation entropy across all timescales of interest, i.e., the area under the MSE curve^35^. Due to differences between term and preterm groups in the curve structure of multiscale entropy, complexity index was also computed within five frequency bands (gamma, beta, alpha, delta and theta), by summing over the timescales corresponding to the upper and lower frequencies of each frequency band.

#### Statistical analysis

If some sensors were removed by infants, only the data from the remaining sensors that were not removed were included for analysis. One infant removed sensors on their wrists. Statistical analyses were conducted in R (version 4.0.2), on Rstudio (version 1.1). Data from twin pairs were assumed to be independent, because the relatively small number of infants in the sample means that models to account for non-independence may not converge, and the most parsimonious, and reliable, model is preferred^48^.

##### Descriptive statistics

Demographic data were described for each group, using means and standard deviations for continuous variables (birthweight, gestational age, age or corrected age (for preterm infants), anthropometry data on length and weight at 9 month follow up), and numbers with percentages for categorical descriptors (gender, ethnicity, socioeconomic status and twin birth, sitting ability). A summary measure indicating whether a comorbidity of preterm birth was present or absent was obtained. Socioeconomic status was measured using the Scottish Index of Multiple Deprivation collected at the antenatal or neonatal timepoint. As physiological arousal and motor activity are thought to be related^49^, Spearman rank correlation coefficients of mean acceleration and complexity index with behavioural data of repetitive movements and negative affect, using data from SF1 only. To determine if physical characteristics influence movement, Pearson correlations of movement acceleration and complexity index with anthropometry data was obtained.

##### Inferential statistics

Linear mixed-effects models (*lmer* package^50^) and planned orthogonal contrasts (*emmeans* package^51^) were used to test hypotheses. Participant ID was included to model random intercepts (i.e., model the motor outcome (acceleration magnitude, permutation entropy, or complexity index) as randomly distributed according to a normal distribution). Models were built with the step-down procedure^52^ with effects of interest included a priori, and model comparisons assessed at the 95% confidence level. Models were fitted using maximum likelihood to enable selection of fixed effects, and final models fitted using REML^52^. Estimated marginal means with 95% confidence intervals were obtained. Degrees-of-freedom of two-tailed tests were estimated using the Kenward-Roger method.

##### MSE curve profile

Main effects of Sensor location, Preterm birth, Scale factor and Preterm birth x Scale factor interaction were included a priori. Where main effects were present, planned orthogonal contrasts with two-tailed t-tests were conducted to examine the differences in infants born preterm relative to infants born at term^53^. As the study is interested in whether there is a difference at any of the examined scale factors (therefore indicating a difference in the curve structure), in any of the sensor locations, only one hypothesis is being evaluated in planned contrasts and therefore does not require adjustment for comparisons at the selected timescales^54–56^.

##### Complexity index

Preterm birth, Sensor location, Phase (still-face paradigm phase), alongside the Phase x Preterm interaction were included as a priori fixed effects. As there was a main effect of Preterm birth and interaction effect of Preterm birth x Sensor, orthogonal contrasts examined the effect of Preterm at each sensor location. Similarly, no correction for multiple comparisons was applied as the contrasts were planned to investigate the hypothesis of whether there would be an effect of Preterm or Phase, at any sensor location.

##### Mean acceleration

The same procedure was repeated for mean acceleration to compare the specificity of the effects on complexity.

##### Exploratory analyses

Due to differences in the curve structure of MSE, in order to specifically explore how Preterm and Phase affects differences in complexity index, complexity index was further split into the five frequency bands before building a linear mixed-effects model for complexity index within each frequency band. As a main effect of Phase was identified for CI in the Gamma, Alpha and Beta band, orthogonal contrasts of the effect of each phase relative to the previous phase was obtained (at the mean across all sensor location and preterm groups). Similarly, Preterm x Sensor effects were observed in CI of all frequency bands except the alpha band, and orthogonal contrasts examined the effect of Preterm at each sensor location for the four frequency bands (at the mean across all phases). Five hypotheses were examined in this independent analysis (that, at one or more sensor locations, there would be an effect of either Phase or Preterm, on complexity in the gamma, beta, alpha, theta and delta frequency bands). Bonferroni correction was applied to correct for the experiment-wise error rate and the null hypothesis was rejection if p_adj_ < 0.05.

##### Power and sample size

This was a proof-of-concept study aiming to recruit at least 10 infants each in the term and preterm group. No power or sensitivity analyses were conducted.

### Study 2

#### Participants

For Study 2, caregiver-infant dyads were included if they participated in their 9-month follow-up appointment before March 2021 and analysable video data was obtained. Study 2 comprised 111 infants (term N = 61; preterm N = 50) who provided analysable behavioural data in the still-face paradigm. The sample is described in detail in a separate publication (Chua et al., in review^39^).

#### Procedure

##### Observational coding of behaviour

Behavioural data on emotional self-regulatory behaviours during the two still-face phases were obtained through an observer coding five types of behaviours (oral-tactile self-comforting, attentional distraction via object manipulation, social interactive, motor stimulatory, and distancing) second-by-second.

##### Chromatic auto-recurrence quantification analysis

Following observational coding, each 120s timeseries comprising infants’ self-regulatory behaviours during each still-face phase (see Fig. S3) was analysed for behavioural dynamics using Chromatic-RQA^29,57^. RQA characterises dynamics of a time series using recurrences, which describe the revisiting of a previous state in time. Recurrences are plotted graphically in a recurrence plot (Fig. S4). Measures can then be obtained to quantify the graphical patterns in a recurrence plot, including specific to recurrences of emotional self-regulatory states (Fig. S5). Detailed procedures for conducting chromatic RQA, including details on specifying parameter settings, are provided in the Supplementary material (Sect. 1.2.1 and Table S1).

#### Measures

##### Emotional self-regulation microlevel behavioural dynamics

Measures of microlevel behavioural dynamics specific to emotional self-regulatory (ER) behavioural states, obtained from Chromatic-RQA, were analysed. Here we provide both the mathematical definition and the contextual interpretation when applied to behaviours in the still-face paradigm. Recurrence rate (RR) is defined as the overall percentage of recurrent points in the recurrence plot (the extent that infants repeat a behaviour). Laminarity (LAM) is the percentage of recurrent points forming vertical lines in the recurrence plot (when behaviours occur, whether they tend to occur briefly, or for a substantial amount of time – defined as 3 s or longer in this study). Low laminarity means that the recurrence plot contains relatively more single recurrent points (or short periods of recurrence), than vertical lines (‘*laminar* states’) that indicate periods of stability. Trapping time (TT) is the average length of vertical lines, indexing the average time spent in a stable state (the average time that bouts of behaviours occur for). Entropy (ENTb) was the average amount of Shannon information in the rectangular block structures present in the RP^58^, and represents the complexity in the temporal patterns in which ER behaviours occurred (whether infants use bouts of behaviours lasting specific lengths of time, or a mix of bouts of varying lengths). We would expect a well-regulated infant to show high recurrence rate (suggesting that behaviours that have occurred are useful for emotion regulation and hence are used again), laminarity (suggesting that these behaviours are stable and can persist for a sufficient length of time to have an effect on emotion), entropy (suggesting that behaviours are used in a flexible manner over time, rather than in the same patterns), and a moderate trapping time (behaviours persisting for stable periods of time, but do not persist for extensive periods which may suggest that the behaviour is not flexible to change).

##### Preterm birth

Preterm birth was assessed using a binary measure of gestational age (preterm: < 33 weeks gestational age; term-born controls: > 37 weeks gestational age). The dose–response effect of preterm birth was assessed using gestational age in weeks. Birthweight Z-scores were used to assess the effect of fetal-growth restriction in both groups, which impacts emotional outcomes^59^, and is often comorbid with preterm birth^60^.

##### Other variables

Proportion of time spent expressing negative affect was obtained as an additional descriptor of behavioural data, which had previously been observationally coded and reported^47^ using the Infant and Caregiver Engagement Phases, revised Heidelberg version^61,62^. Total time spent in any emotional regulation state was expressed as proportion of time, out of 120s.

#### Statistical analyses

All statistical analyses were performed in R (version 4.02), on RStudio (version 1.1). Spearman rank correlation coefficients were obtained to describe the relationship between ER behavioural dynamics with negative affect and total ER behaviours, separately for SF1 and SF2. A repeated-measures, between-subject design was used to examine the effect of preterm birth and the effect of a repeated stressor microlevel behavioural dynamics, using linear mixed effect models (*lmer* package^50^). Power sensitivity analyses using *simr* (version 1.0.5) were carried out to simulate the power for a range of unstandardized interaction and main effect sizes, and then from the simulations we identified the smallest effect sizes that our sample had close to 80% power to detect. Table S2 shows that models for RR and LAM were sensitive to unstandardized effect sizes of < 1%, but were only sensitive to large interaction effects (10% or 6%). While models were only sensitive to effects of 1–4 bits/bin for ENTb and 0.5–2.5s for TT, there is no prior work to support interpretation of whether able to detect any smaller differences would be clinically meaningful. We provide methodological details of interest to specialised readers in the Supporting Information: Sect. 1.2.2 and Fig. S6-S10 on model building and diagnostics and Sect. 1.2.3 on surrogate analyses. Section 1.2.4 describes an exploratory analysis using total emotion regulation behaviours as an outcome measure with results in Table S3 and Fig. S11 to S13.

## Results

### Descriptive statistics

Proportion of time spent using repetitive movements was correlated with mean acceleration of torso (rho = 0.45, p = 0.037), left ankle (rho = 0.58, p = 0.005) and right ankle (rho = 0.50, p = 0.017) movements. There were no correlations between mean acceleration with negative affect, height or weight, and no correlations between complexity index and negative affect or repetitive movements, or height and weight (Table S4).

### Multiscale entropy (MSE) curve profile

**Fig. 2:**
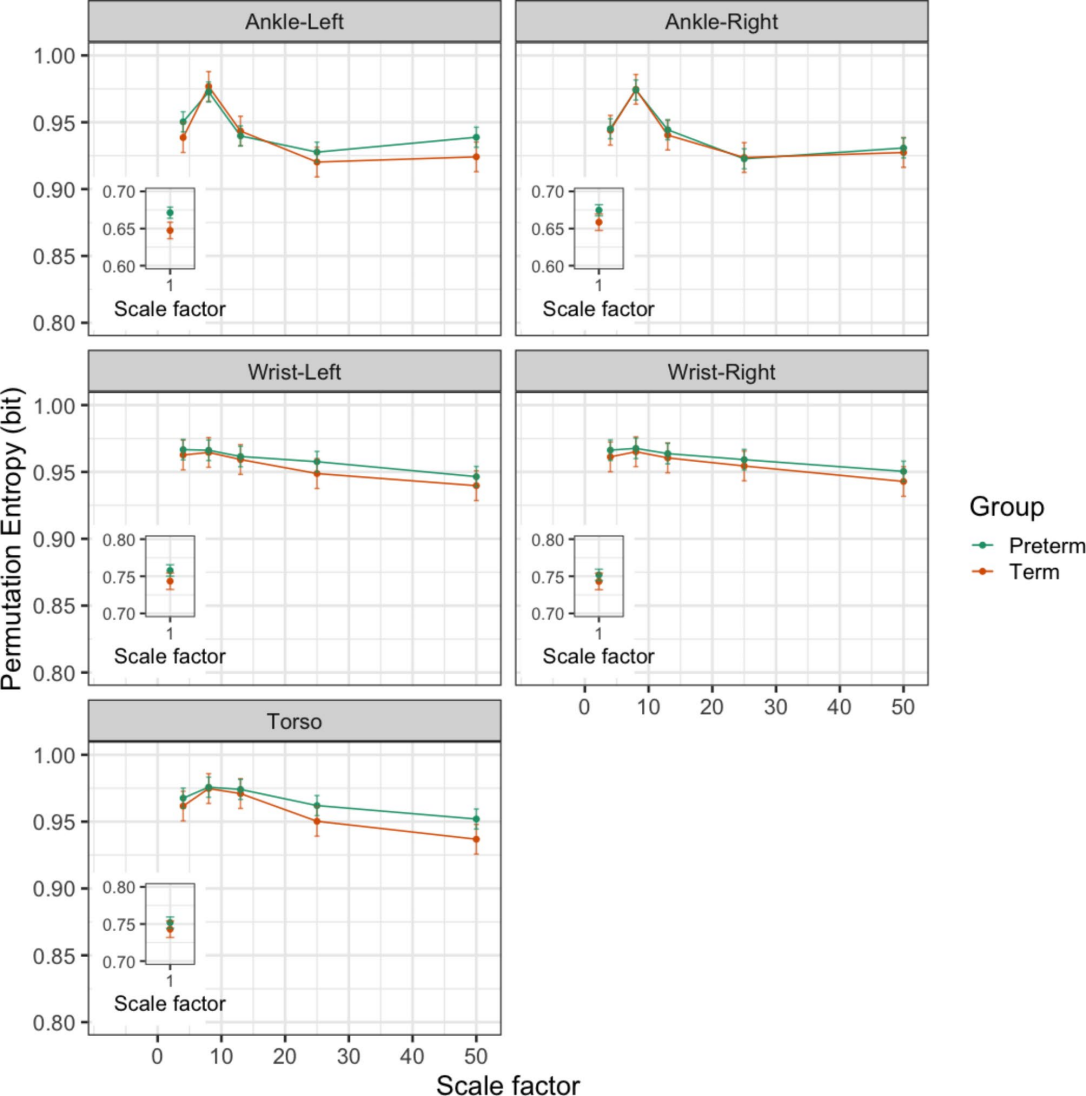
Multiscale Permutation Entropy over 50 scale factors for movement of the left and right ankle, left and right wrists and torso, by preterm and term group. Permutation entropy for scale factor 1 shown in inset, with a different scale for the y-axis.

To examine the curve structure of multiscale permutation entropy, permutation entropy over 50 timescales in the first still-face phase was analysed for effects of Preterm birth, Scale factor, and Sensor location. Scale factor can be analogised to a “zooming out” factor in optical cameras. Zooming out from the signal by *n* times enables us to study activity at a slower frequency differing by a factor of *n*. For example, for scale factor of 50 for a 100Hz sampling frequency, we examine activity at 2 Hz—equivalently, an interval between samples of 0.5s (*timescale)*. Scale factor (F_5, 882.0_ = 4492.65, p < 0.001), Preterm birth (F_1, 29.8_ = 9.33, p = 0.005), Sensor location (F_4, 883.8_ = 157.38, p < 0.001), and Scale x Preterm (F_5, 882.0_ = 4.44, p < 0.001), and Scale x Sensor (F_20, 882.0_ = 29.98, p < 0.001) (Table S5), were predictors of the curve structure of permutation entropy in SF1. Figure 2 shows that the curve structure of multiscale entropy is very similar for both groups between scale factor 8 and 13, but diverges at lower or higher scale factors for the left ankle and torso, with the term group showing lower levels of entropy. The curve for the right ankle, and both wrists are similar across all scale factors, with weak evidence that entropy is lower in the preterm group at scale factor 1.

### Complexity index

Complexity index, the sum of permutation entropy over all 50 timescales, was analysed for effects of Preterm birth, Phase, Sensor location. There were main effects of Preterm (F_1, 29.2_ = 10.36, p = 0.003), Sensor (F_4, 681.8_ = 250.58, p < 0.001) and an interaction effect of Preterm x Sensor (F_4, 681.8_ = 3.71, p = 0.005), but no effect of Phase (F_4, 685.7_ = 1.11, p = 0.353), or Phase x Preterm (F_4, 685.7_ = 0.93, p = 0.447) on complexity index. Table S6 shows the results of contrasts of preterm birth on complexity index and mean acceleration, at the level of each Sensor location. Contrasts identified that the preterm group showed a higher complexity index relative to the term group in movements of the torso (Difference = 2.58 95%CI 1.19–3.98) and both ankles, but no differences in movement of the wrists. A greater difference in complexity index was seen for the left ankle (Difference = 3.04 95%CI 1.65–4.44), than the right ankle movements (Difference = 1.52 95%CI 0.21–2.91).

### Mean acceleration

For comparison with Complexity Index, the same model was run with mean acceleration. For mean acceleration, there was an effect of Sensor (F_4, 687.3_ = 55.44, p < 0.001) and Preterm x Sensor (F_4,687.3_ = 6.80, p < 0.001), but no Preterm effect (F_1, 30.5_ = 1.93, p = 0.174) in the reference sensor location, or effects of Phase (F_4, 689.9_ = 1.29, p = 0.272) or Phase x Preterm (F_4, 689.9_ = 1.24, p = 0.291). Contrasts examining the main effect of Preterm showed that the preterm group showed lower mean accelerations compared to the term group in movements of the ankles, but not the torso or wrists (Table S6). Similar differences were observed for both left (Difference = -0.62 m/s^2^ 95%CI -1.07 – -0.17) and right ankle (Difference = -0.66 m/s^2^ 95%CI -1.11 – -0.21) movements.

### Complexity index within specific frequency bands

Due to the differences in the multiscale entropy curve profile, complexity index was explored within the gamma (30-45Hz), beta (14-30Hz), alpha (8–13.5Hz), theta (4.5–7.5Hz), delta (0.5-4Hz) (in order of higher to lower frequency) as exploratory analyses, with Bonferroni correction applied for five hypotheses tests. Where there was evidence of main effects of Preterm and Phase, planned orthogonal contrasts with two-tailed t-tests were conducted to examine the main effects at the level of the other or level of sensor location.

**Fig. 3:**
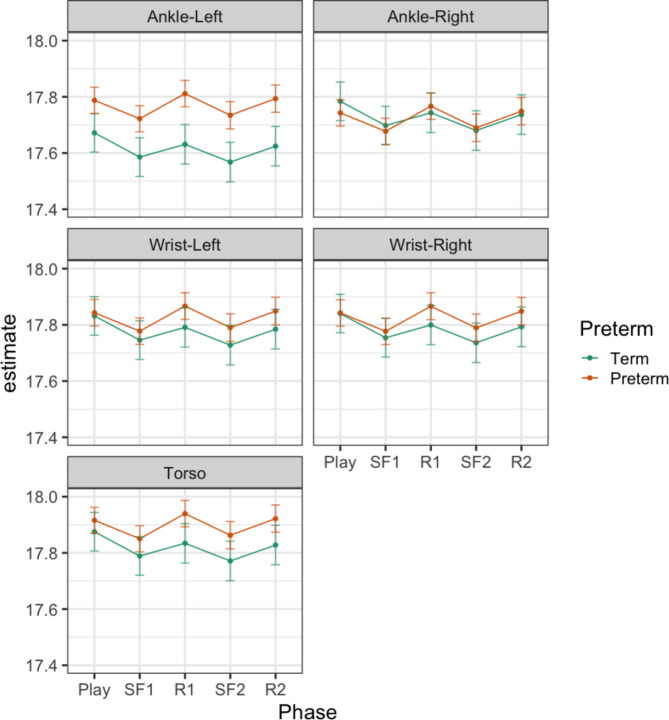
Model results—CI (beta). Predicted margins of complexity index with confidence intervals. Across all sensor locations, complexity index decreases moving from presence to absence of caregiver interaction, and increases moving from absence to presence of caregiver interaction. In this frequency band, complexity index was greater complexity index for infants in the preterm relative to term group, only in left ankle movements.

**Fig. 4:**
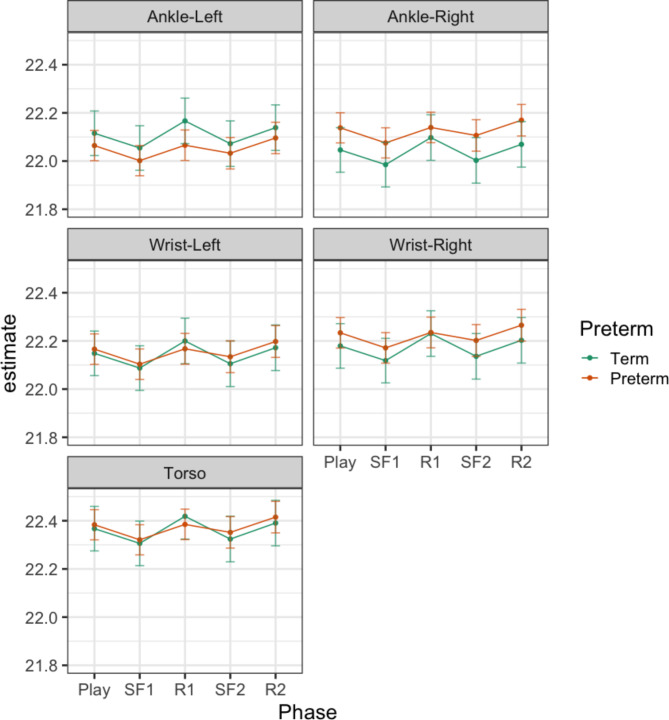
Model results – CI (alpha). Predicted margins of complexity index with confidence intervals. Across all sensor locations, complexity index decreases moving from presence to absence of caregiver interaction, and increases moving from absence to presence of caregiver interaction. In this frequency band, there were no differences between groups in the complexity index.

**Fig. 5:**
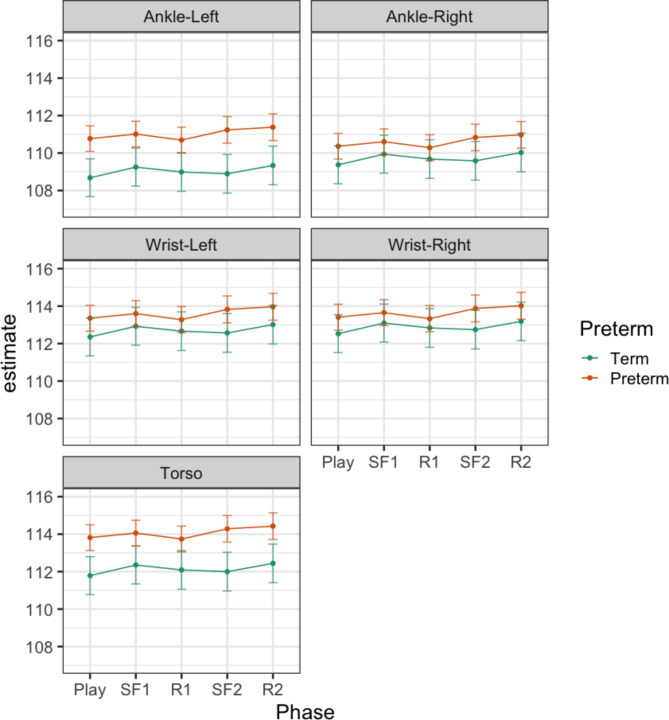
Model results – CI (delta). Predicted margins of complexity index with confidence intervals. In this frequency band, complexity index was greater complexity index for the preterm group compared to the term group, in the left ankle and torso movements. There were no differences between phases with or without caregiver interaction, across all sensor locations.

Table S7 shows the results of the models of complexity index in specific frequency bands. There was an effect of Phase on complexity index in the higher frequency bands (Gamma, Beta and Alpha), but not in the Theta and Delta band. There was an effect of Preterm or Preterm x Sensor in all frequency bands except the Alpha band. There was no effect of Phase x Preterm. Table 2 presents the contrasts examining the main effects of Phase and Preterm in the relevant frequency bands. The difference from one phase to the next presented in Table 3 can be clearly seen in the Beta (Fig. 3) and Alpha (Fig. 4) band, particularly in the Beta band. Differences between infants born at term or preterm age were seen for left ankle movements, in all frequency bands except the Alpha band, and for torso and right ankle movements, only in the low frequency Delta band (Fig. 5). Predicted margins for Gamma and Theta band complexity index are presented in Fig. S14 and S15.

**Table 2:** Contrasts of effect of Phase and effect of Preterm birth on complexity index in the Gamma, Beta, Alpha, Theta and Delta bands. Difference with 95% confidence intervals.

	CI Gamma	CI Beta	CI Alpha	CI Theta	CI Delta
Contrasts of Phase					
SF1 – Play	-0.13 ** **(-0.20 – -0.06)**	-0.08 *** **(-0.11 – -0.04)**	-0.06 * **(-0.11 – -0.02)**	-	-
R1 – SF1	0.05 (-0.02 – 0.12)	0.07 ** **(0.03 – 0.10)**	0.09 ** **(0.04 – 0.13)**	-	-
SF2 – R2	-0.09 (-0.16—-0.01)	-0.07 ** **(-0.11 – -0.03)**	-0.06 * **(-0.11 – -0.02)**	-	-
R2 – SF2	0.10 * **(0.03 – 0.17)**	0.06 ** **(0.02 – 0.09)**	0.06 * **(0.02 – 0.11)**	-	-
Contrasts of Preterm – Term at level of sensor					
Torso	0.20 (0.02 – 0.37)	0.08 (0.01 – 0.15)	-	*0.37 #* *(0.08 – 0.65)*	1.93 ** **(0.84 – 3.03)**
Wrist-Left	0.08 (-0.10 – 0.26)	0.05 (0.02 – 0.12)	-	0.21 (-0.08 – 0.50)	0.90 (-0.20 – 2.00)
Wrist-Right	0.13 (-0.05 – 0.30)	0.04 (-0.03 – 0.11)	-	0.14 (-0.43 – 0.14)	0.78 (-0.32 – 1.88)
Ankle-Left	0.38 *** **(0.20 – 0.56)**	0.15 *** **(0.08 – 0.23)**	-	0.58 ** **(0.29 – 0.86)**	1.99 ** **(0.89 – 3.08)**
Ankle-Right	0.19 (0.01 – 0.36)	0.00 (-0.07 – 0.07)	-	*0.36 #* *(0.07 – 0.64)*	0.89 (-0.20 – 1.98)

^#^p_adj_ < 0.1, *p_adj_ < 0.05 , **p_adj_ < 0.01, ***p_adj_ < 0.001.

Significant values are in [bold].

### Sensitivity analyses

In sensitivity analyses, we restricted the analysis to infants in both groups who could sit unsupported (n = 25) (see Table S8 for sample characteristics). Effects reduced slightly, but remained robust, with the exception of left ankle differences in complexity index in the delta band (Table S9 and S10 and Fig. S16 to S20). However, failure to reach statistical significance for differences in the delta band may also be due to substantially reduced power, given the larger standard error in calculating entropy at longer timescales due to signal coarsegraining (see Methods). Fig. S17 also shows that infants born preterm have greater complexity in right ankle movements in the alpha band, which was not detected in Bonferroni corrected ANOVA analysis of main effects (Table S9), or in the main analysis.

### Study 2

Dynamic measures of emotional self-regulation, recurrence rate (RR), entropy (ENTb), laminarity (LAM) and trapping time (TT) were negatively correlated with negative affect in SF2, with LAM also correlated with negative affect in SF1 (Table S11). All dynamic measures were highly correlated with the total time spent using emotional regulation behaviours (Table S12).

MODELS A examined the effect of Still-face phase (still-face 2 vs still-face 1) and Preterm birth. No effect of Still-face phase or Preterm birth on micro-level behavioural dynamics was found in MODELS A (see Table 3 and Fig. S21).

**Table 3:** MODELS A – effect of prematurity on ER dynamics.

	RR (%)	LAM (%)	ENTb (bits/bin)	TT (s)
	Beta (95% CI)	Beta (95% CI)	Beta (95% CI)	Beta (95% CI)
Fixed effects				
(Intercept)	17.483 *** (14.580 – 20.386)	79.207 *** (75.999 – 82.415)	2.547 *** (2.430 – 2.665)	4.164 *** (3.639 – 4.690)
SF2 [Ref: SF1]	0.101 (-2.097 – 2.299)	-0.515 (-3.738 – 2.708)	-0.032 (-0.141 – 0.078)	-0.091 (-0.563 – 0.382)
Preterm [Ref: Term]	-3.394 (-7.471 – 0.683)	0.142 (-4.119 – 4.403)	-0.130 (-0.289 – 0.029)	0.280 (-0.438 – 0.997)
Random effects				
σ^2^	61.63	135.37	0.16	2.88
τ_00_	84.48 _ID_	55.58 _ID_	0.09 _ID_	2.09 _ID_
ICC	0.58	0.29	0.38	0.42
Marginal R^2^/Conditional R^2^	0.019/0.586	0.000/0.291	0.018/0.391	0.004/0.423

N = 111, Observations = 206.

^#^ p < 0.1 * p < 0.05 ** p < 0.01 *** p < 0.001.

MODELS B examined the effect of Still-face phase and Gestational age. Similarly, no effect of Still-face phase was found in MODELS B in either the term or preterm group (Table S13 and S14; Fig. S22). In the preterm group, gestational age was not associated with LAM and ENTb after removing one extreme data point that was strongly influential (Effect on LAM: 1.31% 95% CI -0.25 – 2.88, p = 0.105; Effect on ENTb: 0.06 bits/bin, 95% CI -0.01 – 0.12, p = 0.087). There was no effect of Birthweight Z-score on micro-level behavioural dynamics (see Table S15 and Fig. S23).

## Discussion

Study 1 is the first proof-of-concept study deploying motor sensors in the still-face paradigm, enabling a fine-grained analysis of motor kinematics across multiple timescales. Computational analyses of these motor patterns revealed signatures in infants’ movement entropy that correspond to different still-face contexts (play, still-face and reunion). Moreover, we found differences between term and preterm groups in movement entropy in left ankle and torso movements. These differences did not change substantially, and remained robust, in sensitivity analyses including only infants who were able to sit unsupported at the time of assessment. In Study 2, we coded and analysed micro-level behavioural dynamics in the still-face phases of the still-face paradigm, but with a larger sample as it is compatible with the standard data collection approach in the still-face paradigm, using video recordings as opposed to wearable sensor data collection. Micro-analysis of the dynamics of behaviour did not reveal differences as a result of repeated stress, and there was no compelling evidence of an effect of preterm birth.

We demonstrated the feasibility and acceptability of using wearable sensors with 9-month-old infants. Infants may find sensor clothing uncomfortable, particularly wearing something over the head. We initially trialled the use of headbands with two infants, who were both significantly distracted by the headband and removed it. Only one infant removed the wrist sensors and some infants interacted briefly with the brightly coloured wristbands and sensor pockets on the socks. None of the infants who had sensors hidden under a long-sleeved shirt were distracted by the wristbands.

Deploying sensors within the still-face paradigm enabled insight into movement and behaviour concurrently. However, sensors, like other objects (e.g. toys, soothers) introduced to the still-face paradigm, may contribute to attentional distraction during stress. This can affect the validity of the paradigm in investigating some research questions-including infants’ stress response, which it was primarily designed to study – if the presence of sensors biases the effect in a particular direction and was not controlled for.

### Strengths and limitations

We used the still-face paradigm not just as a setting related to contextual socioemotional changes, but a setting where infants are able to move freely in a seated position. All infants were able to sit with support or unsupported, but in retrospect sampling criteria could have been stricter to infants who could sit unsupported, as a substantial proportion of infants born preterm were not yet sitting unsupported. Nevertheless, our findings of preterm and term group difference were robust in sensitivity analyses which included only on infants who were able to sit unsupported for at least a minute.

We have focused on differences between infants born at preterm or at term age in motor complexity, recognising that there are multiple explanations why differences may arise, in particular existing research highlight the influence of motor ability, which differs between term and preterm groups, on motor complexity. Given our small proof-of-concept sample, we did not control for motor skills or compared infants with different sitting ability to explore this further, but recommend that future work focus on understanding mechanisms in relation to motor development. Other factors that may affect the development of infants born preterm include poor oxygenation, lung development, and feeding issues, linked to the experience early-life medical complications^46^.

Findings from Study 1 require replication in larger, representative samples. Our sample had more boys than girls in the preterm group. Further investigation is needed on whether there are developmental sex differences in entropy measures, which could have influenced the findings here. Nevertheless, in multi-national studies, sex differences were not found in the attainment of developmental milestones in infancy^63,64^. We explored infant movement across phases of the still-face paradigm but could not differentiate effects specifically due to changes in stress or social interactive context. Although we demonstrated the use of two different dynamic approaches, suitable to the motor kinematic and behavioural data, the results of the two approaches were not directly comparable due to the different methods used. We analysed permutation entropy due to the robustness of this method to measurement and dynamic noise, as well as its multiscale applications, but RQA may also be applied to motor kinematic data. Finally, we did not compare infant behavioural dynamics between the caregiver-unresponsive and caregiver-responsive phases, as the latter requires methods that capture co-regulatory behavioural states, and the resulting co-regulatory states may not be directly comparable with infant self-regulatory behavioural states.

Study 2 requires some consideration of methodological limitations that may have affected our ability to detect group or contextual effects on behavioural dynamics. Although RQA can be applied to short timeseries as we have done here, each two-minute phase may not contain enough of our coded behavioural events to reliably quantify the dynamics of self-regulatory behaviours. We analysed the overall dynamics across all the behavioural sequences regardless of behavioural type, which may have different dynamics^29^. This may have decreased the signal-to-noise ratio in the analysis, occluding potential differences.

Taken together, both studies present several methodological strengths, of note, a focus on different levels of analysis and an emphasis on dynamics aspects of behaviour. In Study 1, we provided behavioural alongside kinematic measures that enabled us to provide evidence to guide the interpretation of complexity index in infancy. The lack of a correlation between complexity index with mean acceleration suggest that it does not simply reflect the amount of motor activity. Mean acceleration did not show differences between phases. Infants born preterm showed lower motor activity indexed by mean acceleration in both ankles, but lower complexity index was found only in the left ankle and torso. Study 2 demonstrates the feasibility of an analysis of behavioural dynamics, alongside traditional approaches used in the still-face paradigm which analyse the amount of behaviours^39^.

Importantly, in Study 1, we advance the application of multiscale entropy. Earlier studies have focused only on the first few Scale factors^65^, and because movement was measured at a high sampling rate, the dynamics at those Scale factors correspond to high frequency activity. We demonstrate novel insights gained from a frequency-specific analysis of movement complexity including lower frequency bands. Complexity index was originally developed as a measure of overall signal complexity, by summing multiscale entropy across all timescales. Mapping the gamma, beta, alpha, delta and theta frequency bands onto the movement complexity curve retained the broad features of the multiscale entropy curve, and frequency-specific complexity index revealed contextual effects and developmental group differences. This approach extends frequency analysis common in analyses of neural Electroencephalography, and muscular Electromyography electrical signals^66,67^. It extends the use of multiscale entropy, more widely applied to neural signals^68^, to motor signals.

### Complexity and regularity at the level of kinematics and behaviour

Earlier studies proposed that lower complexity may reflect stereotypical motor activity^18,19^, although they had not measured the latter. We show here that complexity index was not correlated with repetitive movements. These studies tend to refer to the presence of stereotyped behaviour (e.g. repetitive movements), rather than stereotypy at the kinematic level. Repetitive movements need not have stereotypical kinematics – repetitions of the same movement at the behavioural level can be executed without repetitions at the kinematic level^69^. Movement entropy of 9-month-old infants was also higher in a behaviourally-constrained repetitive rattle-shaking task, than in a behaviourally-unconstrained free play task^24^.

### Psychophysics of entropy in infant development and stress

Entropy has typically been used to quantify movement complexity, referring to variations in temporal patterns of movement. Fundamentally, it is an information-theoretic quantity measuring the information content of the signal. Greater entropy reflects a more disorderly and unpredictable signal, in other words, a signal with a higher information content. In addition to greater movement complexity, greater noise (i.e. random error, such as in measurement; or random disturbances of the motor signal such as neural noise attributed to random firing of cortical neurons or subcortical motor neurons) also contributes to greater values of entropy. However, it is unlikely that greater noise drove our findings of greater entropy in the ankle and torso movements of infants born preterm, as we should see the same pattern of differences across all the sensor locations. It is therefore appropriate to interpret the effects here as characteristic of meaningful differences in movement complexity.

Existing literature support three mechanisms influencing movement complexity, as captured by entropy: contextual effects, motor development and developmental risk. In line with the first mechanism, we found an effect of still-face phase on movement complexity. Changes in emotional context can affect motor processing such as via affecting response inhibition^70^, while changes in interactive contexts alters attentional demands which can impact perceptual modulation of movements^25^. In EEG research, beta-band activity has been linked to integrating sensory feedback to movement, during motor preparation and execution to coordinate ongoing movement or recalibrate movement^71,72^, as well as facilitating more automatic motor responses (e.g. approach or avoidance behaviours) in emotional relative to neutral situations^73^. Alpha-band EEG activity is thought to represent action modulation via perceptual information^74^. It is observed in both action execution and observation in infants as young as 8 months^75,76^, as well as in tasks requiring attention to task-relevant information^77^, sustained attention^78,79^, and joint attention with adults^77,80^. Our findings of an effect of Phase on complexity specific to the beta and alpha bands is in line with the transition between phases in the still-face paradigm of higher levels of perceptual monitoring to guide actions and reciprocal social interactions requiring joint attention, and phases in which those are not needed. We did not find evidence that the effect of contextual changes was different between groups, although this needs further examination given our small sample.

Second, the differences we identified between term and preterm group likely reflect developmental differences between the groups, in line with emerging evidence of developmental changes in movement complexity. Although infants born at term age initially showed higher entropy relative to infants born preterm, this pattern reverses after the acquisition of sitting skill^10^. Infants born at term age showed a greater decrease in entropy, indicating less variable and more predictable postural movements, as postural movements that enable successful control become more consistently used^10,13^. Higher entropy of torso movements may therefore reflect less skilled postural control in the preterm, relative to the term group, further supported by evidence of lower gross and fine motor development in infants born preterm. An earlier study also found that infants born preterm showed more varied patterns of muscular activation than infants born at term during reaching^81^. Further research is needed on why differences in postural complexity were seen specifically in the lower delta frequencies, including any links to the development of anticipatory, prospective control^82^. Interestingly, centre-of-pressure movements measured at 5 Hz became less variable (ie. lower complexity) as infants became more practised at a toy-reaching task^10^. This is around the theta and delta band frequency and our findings may relate to differences in anticipatory postural adjustments^83,84^. For leg movements, approximate entropy decrease across the first 9 months of life^17^, and leg activity shows greater determinism^85^. Differences in the higher frequency beta band in left ankle movements may be due to less smooth movement in infants born preterm, supported by evidence of greater randomness of high-frequency fluctuations in the velocity profile in children relative to adults, indicating a less developed kinematic profile^86^. In contrast, movement forms of arm movements tend to become more varied as infants explore strategies enabling sensorimotor integration, and do not develop consistent prospective control or perceptually-guided movements until childhood^87^.

Finally, our findings do not support the effect of preterm birth on “loss of complexity”. Greater complexity in the preterm group is more likely to be attributed to delayed motor development. However, we found that group differences in movement complexity only in left ankle movements. Similarly, an effect of preterm birth on intralimb coordination were only found in the left leg^88^, and effects of preterm birth on cerebral lateralisation have been reported^89^. This suggests that entropy is sensitive to developmental risk factors, even though the patterns of cerebral lateralisation in preterm birth may be adaptive^89^.

### Future directions

The dynamic measures of movement obtained in this study are embedded within a birth cohort study, enabling future work to test hypotheses on the validity of these measures; for example, whether lower gestational age or white matter injury indicating more severe preterm birth, and developmental stage are independently associated with entropy. Structural and functional MRI measures at neonatal assessment and at 5 years follow up are also available, which can be used to explore how movement entropy relates to neural structure and function.

Deploying sensors to study infant movement is a recent advancement that can easily be combined with computational techniques. Previous work using data obtained through traditional optical motion capture have found differences in kinematic form that relate to early life stress^90^, but they are cumbersome in their approach to visually isolate individual movement bouts, and are not readily scalable^91^. Machine learning techniques have been used with motor sensor data, with promising applications such as to monitor physical growth^92^. However, machine learning techniques still typically focus on the aggregate kinematics of individual movement bouts as the unit of analysis^93^. As such, developing such machine learning algorithms require labelling movement periods in sensor data corresponding to individual bouts of movements, and validating these labels through human observation of movement through videos^93,94^, which is extremely time-intensive. In contrast, entropy can applied to big data without time-intensive data collection methods, particularly permutation entropy which has a lower computational demand compared to other entropy algorithms^43^. We have demonstrated the use of sensors in a semi-ecological setting here, but a key challenge in studying movement entropy in real-world setting will be in controlling for contextual differences. Deployment of sensors have led to acquisition of whole-day data on infant movement, but most of the data may eventually be discarded in analysis of periods of corresponding to similar kinds of activity^95,96^.

Examining micro-level behavioural dynamics is a recent and novel approach. Although further validation work is needed to draw implications, micro-level dynamical approaches provide the means to empirically test theories of emotional regulation and in developmental psychology. For example, social reciprocity of expressive action is an important developmental basis for social understanding and the development of co-regulatory capacities^97,98^ and RQA can examine the cross-system dynamics of dyadic regulation^99,100^. Microanalysis of behaviour can be time-consuming^101^ and may benefit from advances in computer vision to automate behavioural coding.

To conclude, we demonstrate the application of dynamic analysis methods at two levels of infant movement, within an established developmental paradigm. We found preliminary evidence of differences due to preterm birth in movement dynamics at the kinematic level, but not the behavioural level. Frequency-specific analyses identified a “still-face effect” in movement complexity and developmental differences that potentially reflect less advanced postural development, differences in motor abilities, and less smooth kicking movements in infants born preterm. We highlighted key considerations in the interpretation of entropy in infancy and stressful contexts. A straightforward interpretation that lower entropy reflects more stereotyped behaviour or developmental risk was not supported. Investigating movement complexity in infancy need to and account for motor development to accurately capture the effect of developmental risk factors. Our findings inform future work on how multiscale entropy can be applied and interpreted in the study of infant development. While multiscale entropy has been applied widely in the field of movement science, micro-analysis of the temporal structure of behaviour is a relatively young field. It can complement analyses on the total or mean levels of behaviour and has potential applications in various psychological and developmental phenomena.

## Electronic Supplementary Material

Below is the link to the electronic supplementary material.

Supplementary Material 1

## Data Availability

Analyses datasets (comprising de-identified demographic and infant characteristics, entropy data, and RQA data) are stored on Edinburgh DataVault (https://doi.org/10.7488/e65499db-2263-4d3c-9335-55ae6d49af2b), and available to researchers subject to the terms of a data access and collaboration policy: https://www.ed.ac.uk/centre-reproductive-health/tebc/about-tebc/for-researchers/data-access-collaboration. All scripts for pre-processing data, obtaining multiscale permutation entropy, conducting RQA analysis, and statistical analyses will be made openly available upon publication on GitHub (https://git.ecdf.ed.ac.uk/jbrl).
