# Supplementary material for: A multi-level analysis of motor and behavioural dynamics in 9-month-old preterm and term-born infants during changing emotional and interactive contexts
Source: Sci Rep. 2025 Jan 6;15:952. doi: 10.1038/s41598-024-83194-w (PMC11704203; doi:10.1038/s41598-024-83194-w)
Supplement: Supplementary file 1 — Supplementary Material 1 [file 41598_2024_83194_MOESM1_ESM.pdf]

Supplementary Information for

A multi-level analysis of motor and behavioural dynamics in 9-month-old preterm and term-born infants during changing emotional and interactive contexts

Yu Wei Chua*, Lorena Jiménez-Sánchez, Victoria Ledsham, Sinéad O'Carroll, Ralf F. A. Cox, Ivan Andonovic, Christos Tachtatzis, James P. Boardman, Sue Fletcher-Watson, Philip Rowe and Jonathan Delafield-Butt

*Corresponding author. Email: yu.wei.chua@liverpool.ac.uk

This PDF file includes:

1. Supplemental methods
2. Tables S1 to S15
3. Figures S1 to S23

1. Supplemental Methods

1.1. Study 1

1.1.1. Data segmentation

The first author watched the video to identify the video time when the first still-face phase started (SF1), when the experimenter instructed the parent to "switch" and stop responding to the infant. The SF1 start time was rounded down to the nearest second, and then the start of the still-face paradigm was calculated as 2 minutes before the SF1 start time. If a Raspberry Pi camera collected video synchronised to sensor recording was available (n=12), video time was directly converted to the sensor sample time by multiplying by the sampling frequency (100hz). If there was no synchronised video, three methods were applied: by identifying the exact start and end time of the sensor recording (when the experimenter is observed to click the record button in the video) (n=6); identifying a movement artefact in the signal of an additional synchronised sensor attached to a clapperboard (n=14).

1.1.2. Filtering

Selection of filter frequency was guided by previous research identifying that almost all the spectral power in human movement is contained within frequencies lower than 20-30 Hz. Each timeseries of acceleration magnitude (for the whole still-face paradigm) was filtered at 10, 20 25 and 30 Hz, and the cut-off spectral power was calculated at each frequency. 90% of the spectral power was contained within 20 Hz. Therefore, to remove high frequency noise or artefactual effects on the acceleration profile, 20 Hz was chosen.

1.1.3. Data reduction strategy

As a large number of scale factors (50 scale factors) were considered, we did not enter all data into statistical analysis, considering a feature-based approach (e.g., slopes across a range of scales[1–3] which focuses on common features in the MSE curve over all individuals[4]. The MSE curve, applied to EEG and physiological research, is often analysed with reference to frequency ranges[5–7]. While Park and colleagues[4] analysed only a single parameter of interest characterising the local maximum of the MSE curve, they approached the discussion of characteristic differences in MSE curve with reference to specific frequency bands. Human brain signals are commonly split into high and low frequencies corresponding to gamma (30-45Hz), alpha (8-13.5Hz), beta (14-30Hz), theta (4.5-7.5Hz), delta (0.5-4Hz) bands and movement is the output of brain processing, leading to the assumption that changes in motor complexity also corresponds to these frequency bands, as well as the approach to analyse complexity index within each frequency band. Visual assessment showed that these 6 scale factors captured characteristic "landmarks" of the MSE curve profile and retains its characteristic curve profile (Fig. S1 and S2), suggesting that the 6 scale factors were suitable to capture the structure of movement complexity.

1.2. Study 2

1.2.1. *Chromatic auto-RQA*

In auto-RQA, the timeseries is compared against itself and a recurrence plot is produced by plotting all the times (on the x-axis) where the system's state matches itself at another time (on the y-axis), or vice versa. This produces repeating patterns of different lengths along the diagonal, horizontal and vertical lines[8]. Chromatic RQA is a modification of the RQA algorithm to distinguish different types of recurrent states of interest to the researcher[9] and involves colour-coding of each recurrence in the recurrence plot according to a chromatic state space (Fig. S4).

The result is a recurrence plot (RP) where patterns of recurrences in different domains (each domain assigned a different colour) can be distinguished and analysed separately.

Categorical behavioural timeseries show a characteristic pattern in the recurrence plot, checkerboard-like patterns formed of rectangular structures[10]. Therefore, the measures selected for this study were related to the vertical structures in the recurrence plot (or, equivalently, horizontal structures, as the recurrence plot is symmetric when a timeseries is plotted against itself).

Auto-RQA depends on a reconstruction of the state space by embedding a time-delayed replica of the original timeseries against itself, if continuous data is used. With categorical data, the state space is already defined by the pre-defined categories; therefore, reconstructing the state space is not necessary. In this case, the behavioural states that were observationally coded defines the behavioural states infants visit during distress. The free parameters normally required by RQA algorithms, delay d and embedding dimension m, take the value of 1, indicating that the original unembedded timeseries were used.

The final parameter, radius r normally specifies how close to points are in the state space to be counted as a recurrence. Chromatic RQA, is a modification of the RQA algorithm to distinguish different types of recurrent states of interest to the researcher[9]. Recurrence was defined as a match of the exact category of self-regulatory behaviour later. As infants may show two behaviours concurrently, partial match of either one of the behaviours at another point in time was considered a recurrence. This is represented in the full chromatic state space in Table S1. Following colour-coding of each recurrence in the RP, measures relating to different types of recurrence could then be computed separately. This enabled us to obtain dynamics specific to recurrences of emotional self-regulatory (ER) behavioural states.

1.2.2. Model building and diagnostics

Due to low percentage of twin pairs relative to the total number of clusters (N=3, 2.7%), model with two random effects (individuals clustered within family) did not converge. Models were therefore run assuming each infant's data was independent[11], with random effects for accounting for repeated measures within individuals. Interaction effects between prematurity and still-face phase were included in models only if it reached statistical significance at the 95% confidence level, following a step-down strategy[12]. Influence diagnostics (Cook's distance) was checked using *influence.ME* (R package Version 0.9-9). regression assumptions of normality and homogeneity of variance were checked.

Cook's distance, obtained using *influence.ME* (R package Version 0.9-9) was used to examine the sensitivity of model estimates when omitting one or more influential data points. No interaction effects between gestational age and still-face phase were identified after consideration of influential data points. Regression assumptions of normality and homogeneity of variance were checked respectively, by visually inspecting q-q plots and plots of residuals against fitted values (Fig. S6 – S10). The variance of residuals appears to be smaller when LAM was high, and larger only at the largest values of RR and TT, indicating greater noise in using observational coding in identifying these extreme values. As mixed-effect models are robust to violations including homogeneity of residual variance, we did not apply a transformation as this would affect the interpretability of the model[13].

1.2.3. RQA Surrogate analyses

Surrogate analysis is approached via "constrained realisation". In other words, surrogate timeseries were generated from the original data in a way that matches the sampling distribution, other than the characteristic being tested[14]. Random shuffling of the original data produces

timeseries of the same total behavioural response and recurrence rate, but destroys the temporal order within the original timeseries. 100 surrogate timeseries were generated and chr-RQA applied to each timeseries. This generates an approximation of the distribution of values expected from a Gaussian process. The null hypothesis that the value of each behavioural dynamics from the original timeseries came from a Gaussian process was then tested using the *casnet* R package (Version 0.2.2)[15]. *Casnet* obtains the rank order probability for the true value (obtained from the original timeseries). A confidence level is applied to determine if the null hypothesis should be rejected, for example at a 0.01 alpha significance level indicates that the original data led to a value more extreme than the end of the surrogate distribution. As three hypothesis tests were conducted for each timeseries (for TT, ENTb and LAM), Bonferroni correction was applied for three tests at the 0.05 alpha level, leading to rejection of the null hypothesis at alpha<0.017. The null hypothesis that the value of TT and LAM came from a Gaussian process was rejected in 100% of infants, for data from both still-face phases. For ENTb, this was 97.5% for the first still-face phase, and 97.1% for the second still-face phase.

1.2.4. Exploratory analyses using total emotional self-regulatory behaviours

Descriptive statistics showed that the overall behavioural response (total of all types of emotional self-regulatory behaviours) was highly correlated with all measures of behavioural dynamics. We investigated the relationship between preterm birth, gestational age and birthweight Z-score on the total emotional self-regulatory behaviours (MODEL D). This enables a crude comparison of whether the micro-level dynamics obtained from the temporal structure of the behavioural response capture different relationships, and not just patterns strongly driven by the total amount of behaviours used. We did not find evidence of an effect of preterm birth or birthweight Z-score on total emotional self-regulatory

behaviours (see Table S4 and Fig. S11 to S13). As micro-level dynamic measures also showed null effects, we were unable to further consider whether dynamic measures of behaviour may provide different information on emotional self-regulation and therefore capture different group differences or differences between still-face phases.

References

1. Escudero, J., Abásolo, D., Hornero, R., Espino, P. & López, M. Analysis of electroencephalograms in Alzheimer's disease patients with multiscale entropy. *Physiological Measurement* **27**, (2006).
2. Lin, P. F. *et al.* Correlations between the signal complexity of cerebral and cardiac electrical activity: a multiscale entropy analysis. *PloS one* **9**, (2014).
3. Tsai, P. H. *et al.* A Novel Application of Multiscale Entropy in Electroencephalography to Predict the Efficacy of Acetylcholinesterase Inhibitor in Alzheimer's Disease. *Computational and mathematical methods in medicine* **2015**, (2015).
4. Park, J. H., Kim, S., Kim, C. H., Cichocki, A. & Kim, K. Multiscale entropy analysis of EEG from patients under different pathological conditions. *Fractals* **15**, 399–404 (2007).
5. Watanabe, E. *et al.* Multiscale Entropy of the Heart Rate Variability for the Prediction of an Ischemic Stroke in Patients with Permanent Atrial Fibrillation. *PLOS ONE* **10**, e0137144 (2015).
6. Takahashi, T. *et al.* Antipsychotics reverse abnormal EEG complexity in drug-naive schizophrenia: a multiscale entropy analysis. *NeuroImage* **51**, 173–182 (2010).

7. Ho, Y. L., Lin, C., Lin, Y. H. & Lo, M. T. The Prognostic Value of Non-Linear Analysis of Heart Rate Variability in Patients with Congestive Heart Failure—A Pilot Study of Multiscale Entropy. *PLOS ONE* **6**, e18699 (2011).
8. Webber, C. L. & Zbilut, J. P. Recurrence Quantification Analysis of Nonlinear Dynamical Systems. in *Tutorials in contemporary nonlinear methods for the behavioral sciences* vol. 94 26–94 (2005).
9. Xu, T. L., de Barbaro, K., Abney, D. H. & Cox, R. F. A. Finding Structure in Time: Visualizing and Analyzing Behavioral Time Series. *Frontiers in Psychology* **11**, (2020).
10. Leonardi, G. A Method for the computation of entropy in the Recurrence Quantification Analysis of categorical time series. *Physica A: Statistical Mechanics and its Applications* **512**, 824–836 (2018).
11. Sauzet, O., Wright, K. C., Marston, L., Brocklehurst, P. & Peacock, J. L. Modelling the hierarchical structure in datasets with very small clusters: A simulation study to explore the effect of the proportion of clusters when the outcome is continuous. *Statistics in Medicine* **32**, 1429–1438 (2013).
12. Zuur, A. F., Ieno, E. N., Walker, N. J., Saveliev, A. A. & Smith, G. M. Mixed Effects Modelling for Nested Data. in *Mixed effects models and extensions in ecology with R* (eds. Zuur, A. F., Ieno, E. N., Walker, N., Saveliev, A. A. & Smith, G. M.) 101–142 (Springer, New York, NY, 2009). doi:10.1007/978-0-387-87458-6_5.
13. Schielzeth, H. *et al.* Robustness of linear mixed-effects models to violations of distributional assumptions. *Methods in Ecology and Evolution* **11**, 1141–1152 (2020).

14. Theiler, J., Eubank, S., Longtin, A., Galdrikian, B. & Doyne Farmer, J. Testing for nonlinearity in time series: the method of surrogate data. *Physica D: Nonlinear Phenomena* **58**, 77–94 (1992).

15. Hasselman, F., Olthof, M. & Cui, J. casnet: A toolbox for studying Complex Adaptive Systems and NETworks (0.2.2). (2022).

2. Tables S1 to S12

Table S1. Full chromatic state space. State 1-5 corresponds to ER behavioural categories defined in the observational coding scheme. States 12, 13, 14 etc corresponds to concurrent behaviours, for example state 1 and 2 (or 1 and 3, or 1 and 4) within the same second. A recurrence is coded as long as another behavioural state in time matches at least one of the behaviours.

TS2 / TS1	1	2	3	4	5	6	12	13	14	15	23	24	25	34	35	45	123	124	125	134	135	145	234	235	345
1	ER						ER	ER	ER	ER							ER	ER	ER	ER	ER	ER			
2		ER					ER				ER	ER	ER				ER	ER	ER				ER	ER	
3			ER					ER			ER			ER	ER		ER			ER	ER		ER	ER	ER
4				ER					ER			ER		ER		ER		ER		ER		ER	ER		ER
5					ER					ER			ER		ER	ER			ER		ER	ER		ER	ER
6						NER																			
12	ER	ER					ER	ER	ER	ER	ER	ER	ER				ER	ER	ER	ER	ER	ER	ER	ER	
13	ER		ER				ER	ER	ER	ER	ER			ER	ER		ER	ER	ER	ER	ER	ER	ER	ER	ER
14	ER			ER			ER	ER	ER	ER		ER		ER		ER	ER	ER	ER	ER	ER	ER	ER		ER
15	ER				ER		ER	ER	ER	ER			ER		ER	ER	ER	ER	ER	ER	ER	ER		ER	ER
23		ER	ER				ER	ER			ER	ER	ER	ER	ER		ER	ER	ER	ER	ER		ER	ER	ER
24		ER		ER			ER		ER		ER	ER	ER	ER		ER	ER	ER	ER	ER		ER	ER	ER	ER
25		ER			ER		ER			ER	ER	ER	ER		ER	ER	ER	ER	ER		ER	ER	ER	ER	ER
34			ER	ER				ER	ER		ER	ER		ER	ER	ER	ER	ER		ER	ER	ER	ER	ER	ER
35			ER		ER			ER		ER	ER		ER	ER	ER	ER	ER		ER	ER	ER	ER	ER	ER	ER
45				ER	ER				ER	ER		ER	ER	ER	ER	ER		ER	ER	ER	ER	ER	ER	ER	ER
123	ER	ER	ER				ER	ER	ER	ER	ER	ER	ER	ER	ER		ER	ER	ER	ER	ER	ER	ER	ER	ER
124	ER	ER		ER			ER	ER	ER	ER	ER	ER	ER	ER		ER	ER	ER	ER	ER	ER	ER	ER	ER	ER
125	ER	ER			ER		ER	ER	ER	ER	ER	ER	ER		ER	ER	ER	ER	ER	ER	ER	ER	ER	ER	ER
134	ER		ER	ER			ER	ER	ER	ER	ER	ER		ER	ER	ER	ER	ER	ER	ER	ER	ER	ER	ER	ER
135	ER		ER		ER		ER	ER	ER	ER	ER		ER	ER	ER	ER	ER	ER	ER	ER	ER	ER	ER	ER	ER
145	ER			ER	ER		ER	ER	ER	ER		ER	ER	ER	ER	ER	ER	ER	ER	ER	ER	ER	ER	ER	ER
234		ER	ER	ER			ER	ER	ER		ER	ER	ER	ER	ER	ER	ER	ER	ER	ER	ER	ER	ER	ER	ER
235		ER	ER		ER		ER	ER		ER	ER	ER	ER	ER	ER	ER	ER	ER	ER	ER	ER	ER	ER	ER	ER
345			ER	ER	ER			ER	ER	ER	ER	ER	ER	ER	ER	ER	ER	ER	ER	ER	ER	ER	ER	ER	ER

Table S2. Results of power sensitivity analysis (micro-level dynamic outcomes). RR and LAM were sensitive to unstandardized effect sizes of <1%, but were only sensitive to larger interaction effects. While models were only sensitive to effects of 1-4 bits/bin for ENTb and 0.5s - 2.5s for TT, it is unclear if being able to detect any smaller differences would be clinically meaningful.

			Effect size detectable with approximately 80% Power			
Model	Sample size	Model term	RR (%)	LAM (%)	ENTb (bits/bin)	TT (s)
A	N=111, observations=206	Preterm x SF	10.0	6.0	>4.0	>5.0
A	N=111, observations=206	Preterm	<1	<1	4.0	2.5
A	N=111, observations=206	SF	<1	<1	1.0 - 2.0	0.5
B1 (term)	N=61, observations=113	SF	7.0	5.0	>4.0	5.0
B2 (preterm)	N=50, observations=93	SF	6.0	4.0	>4.0	4.0
B1 (term)	N=61, observations=113	GA	3.6	3.5	>2.0	>2.5
B2 (preterm)	N=50, observations=93	GA	2.0	2.0	2.0	2.0
C	N=111, observations=206	Birthweight Z-score	3.0	3.0	3.0	3.0

Table S3. MODELS D – effect of preterm birth, gestational age and birthweight Z-score on Total ER behaviours

	Total ER	Total ER	Total ER (Term)	Total ER (Preterm)
**Fixed effects**	**Beta (95% CI)**	**Beta (95% CI)**	**Beta (95% CI)**	**Beta (95% CI)**
(Intercept)	0.567 *** (0.522 – 0.612)	0.549 *** (0.512 – 0.586)	0.579 *** (0.501 – 0.657)	0.291 ** (0.093 – 0.490)
SF [Ref: SF1]	-0.018 (-0.052 – 0.015)	-0.018 (-0.052 – 0.015)	-0.020 (-0.067 – 0.027)	-0.022 (-0.068 – 0.023)
Preterm [Ref: Term]	-0.037 (-0.100 – 0.026)			
Birthweight Z-score		0.009 (-0.022 – 0.041)		
Gestation [weeks]			-0.005 (-0.033 – 0.023)	0.024# (-0.001 – 0.050)
**Random effects**				
σ^2	0.01	0.01	0.02	0.01
τ_{00}	0.02 $_{ID}$	0.02 $_{ID}$	0.01 $_{ID}$	0.03 $_{ID}$
ICC	0.59	0.59	0.44	0.70
Marginal R^2 / Conditional R^2	0.012 / 0.592	0.004 / 0.588	0.006 / 0.439	0.058 / 0.720
N, Observations	111 $_{ID}$, 206	111 $_{ID}$, 206	61 $_{ID}$, 113	50 $_{ID}$, 93

# *p<0.1* * *p<0.05* ** *p<0.01* *** *p<0.001*

Table S4. Spearman rank correlation coefficients between mean acceleration and complexity index with proportion of time using repetitive behaviours, proportion of time showing negative affect, height, and weight of infant. Data from SF1, for subset of infants with observationally-coded behavioural data (N=22).

	Negative affect	Repetitive movements	Height	Weight
Mean acceleration				
Torso	-0.28	0.45 *	-0.01	-0.05
Wrist-Right	-0.07	0.37	-0.15	0.14
Wrist-Left	-0.09	0.29	-0.05	0.04
Ankle-Right	0.08	0.50 *	-0.26	-0.21
Ankle-Left	-0.04	0.58 **	-0.14	-0.15
Complexity Index				
Torso	-0.31	0.04	0.30	-0.07
Wrist-Right	-0.38	0.30	0.41	-0.01
Wrist-Left	-0.40	0.28	0.23	0.01
Ankle-Right	0.02	0.02	0.11	-0.01
Ankle-Left	0.25	-0.10	-0.14	-0.25

$^{\#}p<0.1$ $^{*}p<0.05$, $^{**}p<0.01$, $^{***}p<0.001$

Table S5. ANOVA table of the effect of Scale factor, Preterm birth and Sensor location on Permutation entropy, and Phase, Preterm birth and Sensor location on Complexity Index and Mean Acceleration.

	SS	MS	NumDF	DenDF	F	p
**Curve structure**						
Scale factor	6.60	1.32	5	882.01	**4492.65*****	<0.001
Preterm birth	0.00	0.00	1	29.81	**9.33****	0.005
Sensor location	0.18	0.05	4	883.79	**157.38*****	<0.001
Scale factor x Preterm	0.01	0.00	5	882.01	**4.44****	0.001
Scale factor x Sensor	0.18	0.01	20	882.01	**29.98*****	<0.001
Preterm x Sensor	0.00	0.00	4	858.84	0.84	0.502
Scale x Preterm x Sensor	0.00	0.00	20	857.99	0.40	0.992
**Complexity Index**						
Phase	27.50	6.87	4	685.70	1.11	0.353
Preterm birth	64.40	64.39	1	29.24	**10.36****	0.003
Sensor location	6229.10	1557.28	4	681.76	**250.58*****	<0.001
Phase x Preterm	23.10	5.76	4	685.70	0.93	0.447
Preterm x Sensor	92.30	23.07	4	681.76	**3.71****	0.005
**Acceleration**						
Phase	2.53	0.63	4	689.92	1.29	0.272
Preterm birth	0.95	0.95	1	30.05	1.93	0.174
Sensor location	108.67	27.17	4	687.31	**55.44*****	<0.001
Phase x Preterm	2.44	0.61	4	689.92	1.24	0.291

Preterm x Sensor	13.33	3.33	4	687.31	**6.80***	<0.001

# $p<0.1$ * $p<0.05$, ** $p<0.01$, *** $p<0.001$

Table S6. Contrasts of differences between Preterm group relative to the term group in Complexity Index and mean acceleration, at each sensor location

	Difference (95% CI)	t	Df	p
Contrasts of Preterm – Term at level of Sensor				
Complexity index (total)				
Torso	**2.58** ** **(1.19 – 3.98)**	3.63	60.22	0.001
Wrist-Left	*1.26*# *(-0.14 – 2.66)*	1.76	61.10	0.083
Wrist-Right	1.14 (-0.26 – 2.54)	1.59	61.10	0.116
Ankle-Left	**3.04** *** **(1.65 – 4.44)**	4.28	60.22	<0.001
Ankle-Right	**1.52** * **(0.12 – 2.91)**	2.13	60.22	0.037
Acceleration				
Torso	-0.08 (-0.53 – 0.37)	0.73	49.67	0.726
Wrist-Left	-0.06 (-0.51 – 0.39)	0.80	50.22	0.805
Wrist-Right	-0.01 (-0.46 – 0.44)	0.96	50.22	0.959
Ankle-Left	**-0.62** * **(-1.07 – -0.17)**	2.64	49.67	0.011
Ankle-Right	**-0.66** * **(-1.11 – -0.21)**	2.83	49.67	0.007

# *p<0.1* * *p <0.05* , ** *p <0.01*, *** *p <0.001*

Table S7. ANOVA table of the effect of Phase, Preterm birth and Sensor location, with interaction effects of Phase x Preterm and Preterm x Sensor on Complexity Index within the Gamma, Beta, Alpha, Theta and Delta band. P-values adjusted for five comparisons.

	SS	MS	NumDF	DenDF	F	p	p_{adj}
**CI - Gamma**							
Phase	2.06	0.52	4	685.62	**6.03*****	<0.001	<0.001
Preterm birth	0.53	0.53	1	30.03	6.14$^{\#}$	0.019	0.095
Sensor location	147.76	36.94	4	682.43	**432.16*****	<0.001	<0.001
Phase x Preterm	0.54	0.14	4	685.62	1.59	0.176	0.880
Preterm x Sensor	1.66	0.41	4	682.43	**4.85****	0.001	0.004
**CI - Beta**							
Phase	0.73	0.18	4	686.85	**8.94*****	<0.001	<0.001
Preterm birth	0.10	0.10	1	29.04	4.95	0.034	0.170
Sensor location	2.25	0.56	4	681.81	**27.53*****	<0.001	<0.001
Phase x Preterm	0.09	0.02	4	686.85	1.10	0.357	1.785
Prematurity x Sensor	0.44	0.11	4	681.81	**5.32****	<0.001	0.002
**CI - Alpha**							
Phase	0.79	0.20	4	685.84	**5.97****	0.000	0.001
Preterm birth	0.01	0.01	1	28.92	0.23	0.638	3.189
Sensor location	7.07	1.77	4	681.50	**53.14*****	<0.001	<0.001
Phase x Preterm	0.08	0.02	4	685.84	0.58	0.679	3.396
Prematurity x Sensor	0.36	0.09	4	681.50	2.71	0.029	0.147
**CI - Theta**							
Phase	0.05	0.01	4	688.89	0.04	0.997	4.987
Preterm birth	3.48	3.48	1	30.32	**9.28***	0.005	0.024
Sensor location	644.96	161.24	4	683.32	**429.89*****	<0.001	<0.001
Phase x Preterm	2.98	0.75	4	688.89	1.99	0.095	0.474

Prematurity x Sensor	3.53	0.88	4	683.32	2.35	0.053	0.264
**CI - Delta**							
Phase	31.14	7.79	4	685.01	2.28	0.060	0.298
Preterm birth	25.43	25.43	1	29.07	*7.44*$^{\#}$	0.011	0.054
Sensor location	1451.97	362.99	4	681.49	**106.17*****	<0.001	<0.001
Phase x Preterm	8.34	2.08	4	685.01	0.61	0.656	3.280
Prematurity x Sensor	46.83	11.71	4	681.49	**3.42***	0.009	0.044

$^{\#}p_{adj}<0.1$, $^{*}p_{adj}<0.05$, $^{**}p_{adj}<0.01$, $^{***}p_{adj}<0.001$

Table S8. Clinical and developmental characteristics of infants who could sit unsupported or sit supported.

Characteristic	Term, sits unsupported, N = 10	Preterm, sits unsupported, N = 15	Preterm, sits supported, N = 7
Age or corrected age at visit (months)	8.9 (0.3)	9.0 (0.6)	8.4 (0.5)
Birthweight (g)	3,470.8 (370.6)	1,463.2 (480.4)	1,417.7 (545.0)
Birthweight Z-score	0.5 (0.6)	0.4 (1.1)	-0.4 (1.5)
Gestation (weeks)	39.4 (1.8)	28.9 (2.7)	30.0 (2.2)
Height	71.3 (1.5)	73.0 (3.5)	70.1 (5.3)
Weight	9.3 (1.2)	9.2 (1.1)	7.6 (1.3)
Motor raw score	26.9 (5.4)	25.2 (5.6)	16.0 (2.4)
Gross motor raw score	13.5 (4.2)	12.9 (3.8)	7.4 (2.6)
Fine motor raw score	13.4 (2.5)	12.3 (2.5)	8.6 (2.3)
Any comorbidity of preterm birth	NA	9 (60.0%)	3 (43.9%)

Table S9. Sensitivity analysis, including only term and preterm-born infants who able to sit unsupported (n=25). ANOVA table of the effect of Phase, Preterm birth and Sensor location, with interaction effects of Phase x Preterm and Preterm x Sensor on Complexity Index within the Gamma, Beta, Alpha, Theta and Delta band. P-values adjusted for five comparisons.

	SS	MS	NumDF	DenDF	F	p	p_{adj}
**CI - Gamma**							
Phase	1.10	0.28	4	537.36	*3.20*#	0.013	0.065
Preterm birth	0.40	0.40	1	23.23	4.60	0.043	0.213
Sensor location	139.50	34.87	4	534.82	**404.61***	0.000	0.000
Phase x Preterm	0.95	0.24	4	537.36	2.77	0.027	0.134
Preterm x Sensor	0.63	0.16	4	534.82	1.81	0.125	0.623
**CI - Beta**							
Phase	0.62	0.16	4	537.36	**7.66***	0.000	0.000
Preterm birth	0.05	0.05	1	22.27	2.68	0.116	0.578
Sensor location	1.98	0.50	4	534.10	**24.36***	0.000	0.000
Phase x Preterm	0.08	0.02	4	537.36	1.02	0.396	1.981
Prematurity x Sensor	0.31	0.08	4	534.10	**3.77***	0.005	0.024
**CI - Alpha**							
Phase	0.54	0.14	4	537.55	**5.06****	0.001	0.003
Preterm birth	0.06	0.06	1	22.83	2.12	0.159	0.794
Sensor location	5.02	1.25	4	534.58	**46.68***	0.000	0.000
Phase x Preterm	0.10	0.03	4	537.55	0.96	0.430	2.152
Prematurity x Sensor	0.34	0.08	4	534.58	*3.12*#	0.015	0.074
**CI - Theta**							
Phase	0.26	0.07	4	539.04	0.18	0.950	4.750
Preterm birth	3.60	3.60	1	23.01	**9.66***	0.005	0.025
Sensor location	569.33	142.33	4	535.18	**382.36***	0.000	0.000

Phase x Preterm	4.54	1.14	4	539.04	*3.05*$^{\#}$	0.017	0.083
Prematurity x Sensor	2.71	0.68	4	535.18	1.82	0.124	0.619
**CI - Delta**							
Phase	30.51	7.63	4	536.77	2.24	0.064	0.319
Preterm birth	15.24	15.24	1	22.67	4.47	0.046	0.228
Sensor location	1334.68	333.67	4	534.25	**97.89*****	0.000	0.000
Phase x Preterm	13.04	3.26	4	536.77	0.96	0.431	2.154
Prematurity x Sensor	30.99	7.75	4	534.25	2.27	0.060	0.301

$^{\#}p_{adj}<0.1$, $^{*}p_{adj}<0.05$, $^{**}p_{adj}<0.01$, $^{***}p_{adj}<0.001$

Table S10. Sensitivity analyses, including only term and preterm-born infants who able to sit unsupported (n=25). Contrasts of effect of Phase and effect of Preterm birth on complexity index in the Gamma, Beta, Alpha, Theta and Delta bands. Difference with 95% confidence intervals.

	CI Gamma	CI Beta	CI Alpha	CI Theta	CI Delta
Contrasts of Phase					
SF1 – Play	**-0.11** *	**-0.07** **	**-0.03**		
	**(-0.18 – -0.03)**	**(-0.11 – -0.03)**	**(-0.08 – 0.01)**	-	-
R1 – SF1	0.04	**0.06** **	**0.07** **		
	(-0.04 – 0.12)	**(0.03 – 0.10)**	**(0.03 – 0.12)**	-	-
SF2 – R2	-0.05	**-0.07** **	**-0.07** *		
	(-0.13 - -0.03)	**(-0.11 – -0.04)**	**(-0.11 – -0.02)**	-	-
R2 – SF2	0.08	**0.06** **	**0.07** *		
	(0.005 – 0.16)	**(0.02 – 0.10)**	**(0.02 – 0.11)**	-	-
Contrasts of Preterm – Term at level of sensor					
Torso	0.16	0.07		*0.38* #	**1.75** **
	(-0.01 – 0.34)	(-0.01 – 0.15)	-	*(0.08 – 0.68)*	**(0.62 – 2.88)**

Wrist-Left	0.11	0.03		0.29	0.72
	(-0.07 – 0.29)	(-0.05 – 0.11)	-	(-0.02 – 0.59)	(-0.41 – 1.86)
Wrist-Right	0.13	0.03		0.14	0.52
	(-0.05 – 0.31)	(-0.05 – 0.11)	-	(-0.16 – 0.45)	(-0.62 – 1.65)
Ankle-Left	**0.29** *	**0.13** **		**0.58** **	*1.43* $^{\#}$
	**(0.11 – 0.47)**	**(0.06 – 0.21)**	-	**(0.28 – 0.87)**	*(0.29 – 2.56)*
Ankle-Right	0.12	0.003		*0.37* $^{\#}$	0.73
	(-0.06 – 0.29)	(-0.08 – 0.07)	-	*(0.07 – 0.67)*	(-0.40 – 1.86)

$^{\#}p_{adj}<0.1$, $^{*}p_{adj}<0.05$, $^{**}p_{adj}<0.01$, $^{***}p_{adj}<0.001$

Table S11. Spearman rank sum correlation coefficients between negative affect with ER behaviours, and with ER behavioural dynamics.

	Negative Affect	
	SF1, Rho	SF2, Rho
ER behavioural dynamics		
RR	0.01	-0.22*
ENTb	-0.12	-0.24*
LAM	-0.20*	-0.24*
TT	-0.14	-0.23*

*# p<0.1   * p<0.05   ** p<0.01   *** p<0.001*

Table S12. Moderate and strong Spearman rank sum correlations between the Total ER response with each measure of behavioural dynamics in SF1 and SF2.

	Total ER	
	SF1, Rho	SF2, Rho
ER behavioural dynamics		
RR	0.924^{***}	0.904^{***}
ENTb	0.368^{***}	0.499^{***}
LAM	0.655^{***}	0.750^{***}
TT	0.416^{***}	0.536^{***}

*** *p<0.001*

Table S13. MODELS B (term) – effect of gestational age on ER behavioural dynamics in term-born infants

	RR (%)	LAM (%)	ENTb (bits/bin)	TT (s)
**Fixed effects**	**Beta (95% CI)**	**Beta (95% CI)**	**Beta (95% CI)**	**Beta (95% CI)**
(Intercept)	18.686 *** (12.664 – 24.709)	75.344 *** (69.368 – 81.320)	2.499 *** (2.307 – 2.691)	3.804 *** (2.986 – 4.622)
SF [Ref: SF1]	0.101 (-3.237 – 3.440)	0.171 (-3.772 – 4.114)	-0.078 (-0.213 – 0.057)	0.222 (-0.283 – 0.726)
Gestation [weeks]	-0.529 (-2.709 – 1.652)	1.489 (-0.642 – 3.621)	0.029 (-0.039 – 0.097)	0.093 (-0.201 – 0.387)
**Random effects**				
σ^2	78.03	110.17	0.13	1.80
τ_{00}	83.08 $_{ID}$	60.41 $_{ID}$	0.05 $_{ID}$	1.30 $_{ID}$
ICC	0.52	0.35	0.29	0.42
Marginal R^2 / Conditional R^2	0.003 / 0.517	0.022 / 0.368	0.015 / 0.297	0.009 / 0.426

N=61, Observations=113

*# p<0.1   * p<0.05   ** p<0.01   *** p<0.001*

Table S14. MODELS B (preterm). Effects of gestational age and SF phase on ER behavioural dynamics

	RR (%)	LAM (%)	ENTb (bits/bin)	TT (s)
**Fixed effects**	**Beta (95% CI)**	**Beta (95% CI)**	**Beta (95% CI)**	**Beta (95% CI)**
(Intercept)	7.382 (-2.774 – 17.538)	68.199 *** (56.655 – 79.743)	1.534 *** (0.994 – 2.074)	3.773 ** (1.410 – 6.137)
SF2 [Ref: SF1]	-0.124 (-2.834 – 2.586)	-1.574 (-6.770 – 3.622)	0.007 (-0.166 – 0.179)	-0.495 (-1.316 – 0.326)
Gestation [weeks]	0.946 (-0.414 – 2.305)	1.625 * (0.101 – 3.149)	0.085 * (0.021 – 0.150)	0.118 (-0.196 – 0.433)
**Random effects**				
σ^2	78.03	110.17	0.13	1.80
τ_{00}	83.08 $_{ID}$	60.41 $_{ID}$	0.05 $_{ID}$	1.30 $_{ID}$
ICC	0.52	0.35	0.29	0.42
Marginal R^2 / Conditional R^2	0.003 / 0.517	0.022 / 0.368	0.015 / 0.2§97	0.009 / 0.426

N=50, Observations=93

*# p<0.1 * p<0.05 ** p<0.01 *** p<0.001*

Table S15. MODELS C – effect of birthweight Z-score on ER behavioural dynamics

	RR (%)	LAM (%)	ENTb (bits/bin)	TT (s)
**Fixed effects**	**Beta (95% CI)**	**Beta (95% CI)**	**Beta (95% CI)**	**Beta (95% CI)**
(Intercept)	15.649 *** (13.224 – 18.075)	78.744 *** (76.046 – 81.441)	2.462 *** (2.363 – 2.561)	4.209 *** (3.768 – 4.650)
SF2 [Ref: SF1]	0.033 (-2.189 – 2.256)	-0.421 (-3.670 – 2.828)	-0.032 (-0.143 – 0.078)	-0.086 (-0.561 – 0.389)
Birthweight Z-score	0.969 (-1.202 – 3.141)	1.716 (-0.508 – 3.939)	0.075# (-0.008 – 0.159)	0.253 (-0.125 – 0.632)
**Random effects**				
σ^2	62.34	136.35	0.16	2.88
τ_{00}	86.57 ID	51.47 ID	0.09 ID	2.08 ID
ICC	0.58	0.27	0.37	0.42
Marginal R^2 / Conditional R^2	0.006 / 0.584	0.014 / 0.284	0.021 / 0.386	0.012 / 0.426

N=111, Observations=206

*# p<0.1 * p<0.05 ** p<0.01 *** p<0.001*

Figures S1 to S18

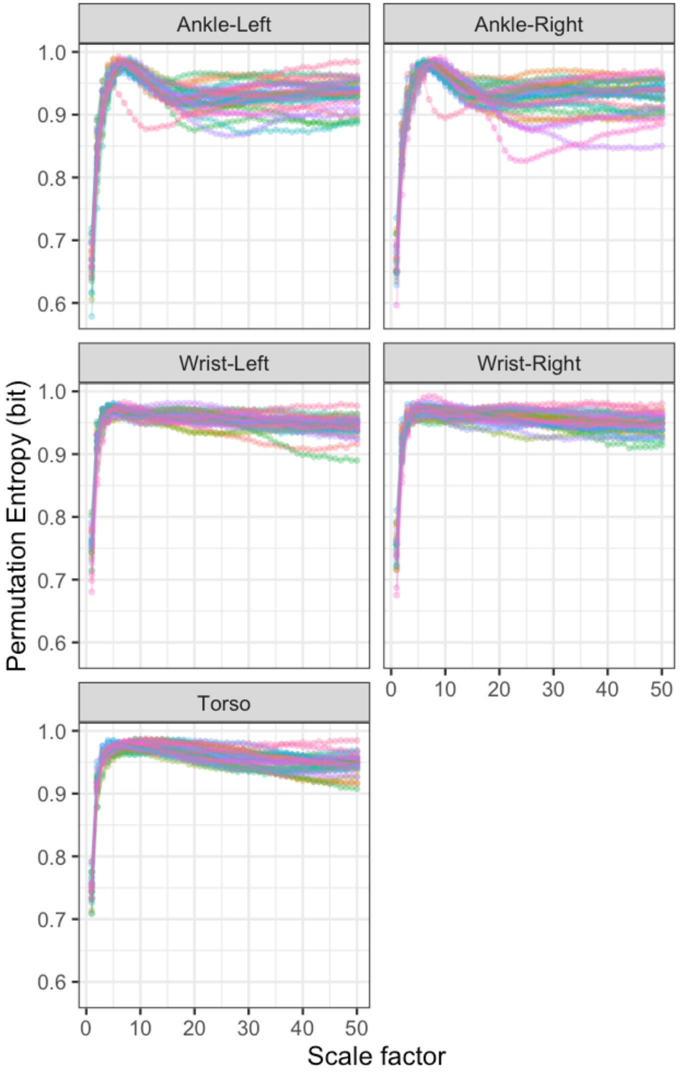

Figure S1. Descriptive plot of multiscale permutation entropy across 50 timescales.

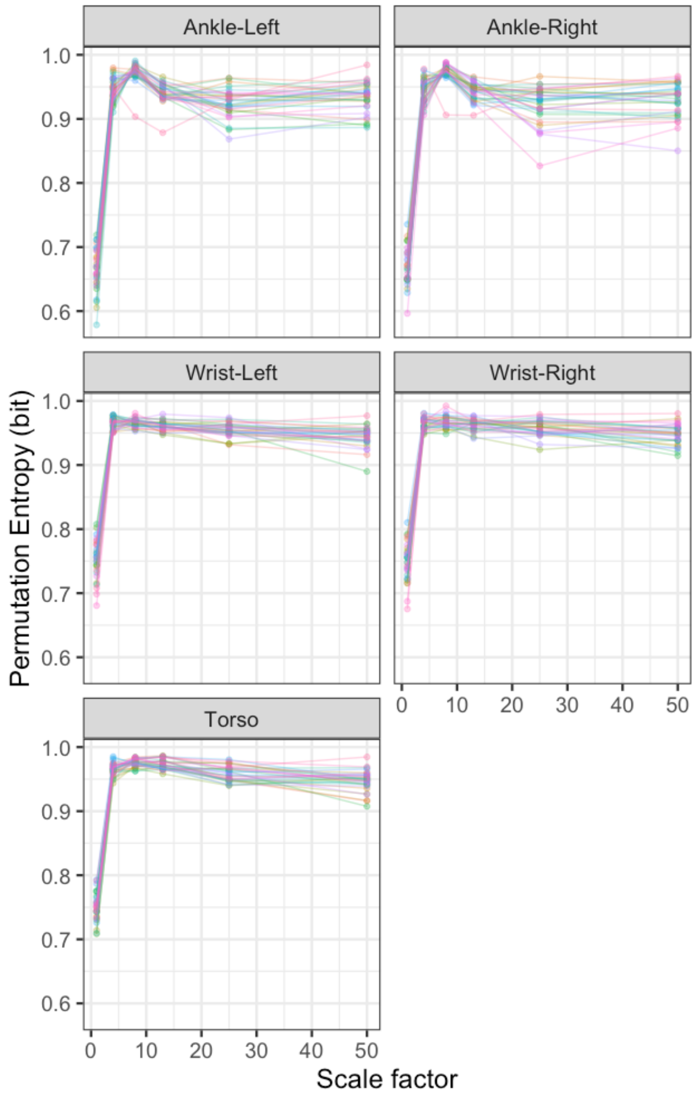

Figure S2. Descriptive plot of reduced dataset of multiscale permutation entropy, 6 scales corresponding to upper and lower frequencies of gamma, beta, alpha, delta, theta bands.

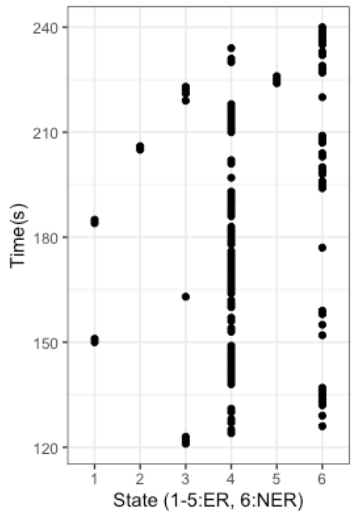

Figure S3. Example of a categorical timeseries of behavioural response during the 120s of the first still-face phase (120 – 240s of the whole still-face paradigm)

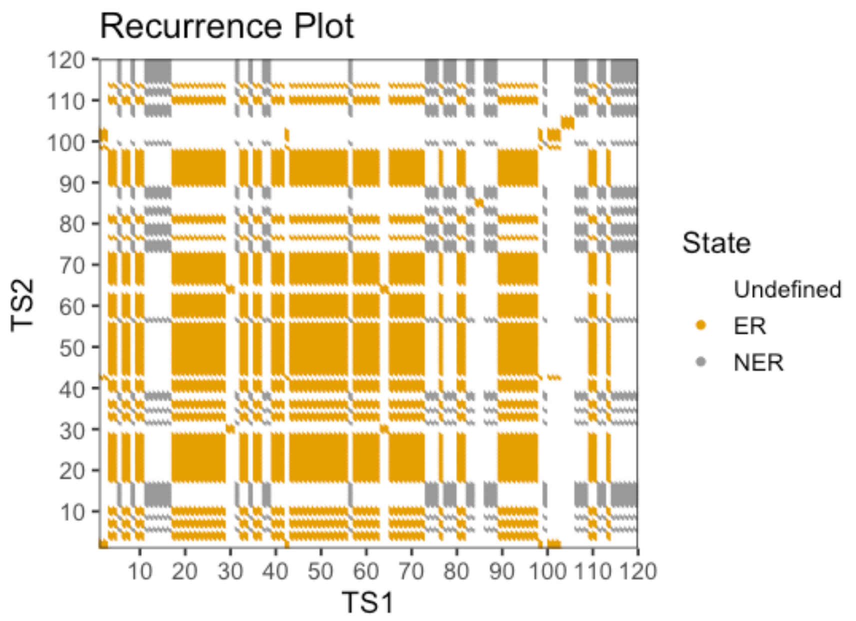

Figure S4. Example of a recurrence plot. Orange dots represent recurrences of ER states, while grey dots represent recurrences of non-ER states.

	1	2	3	4	5	6
1	ER					
2		ER				
3			ER			
4				ER		
5					ER	
6						NER

Figure S5. Simplified chromatic state space. States 1 to 5 correspond to ER states as defined in the video coding scheme (SC, SOC, OBJ, RME and DIST). State 6 corresponds to Non-ER states (MOV: Undefined/Other movement). Recurrences of States 1 to 5 later in time were considered an ER recurrence (coded in Orange). Recurrences of State 6 were considered a non-ER recurrence (coded in Grey). White spaces represent undefined recurrences, not of interest to the present study.

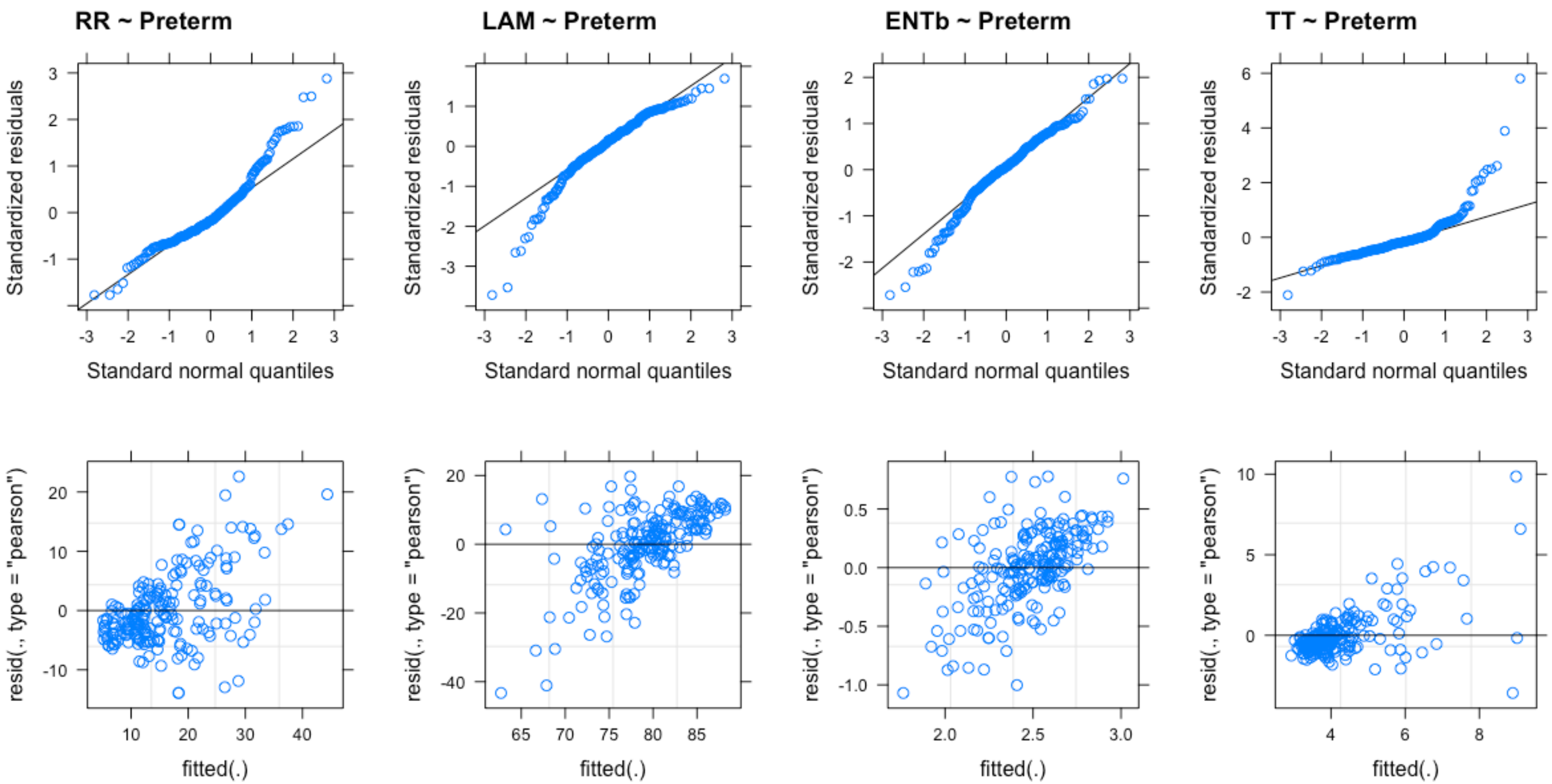

Figure S6. Model A diagnostic plots for behavioural dynamics (top: normal q-q plots, bottom: residuals vs fitted)

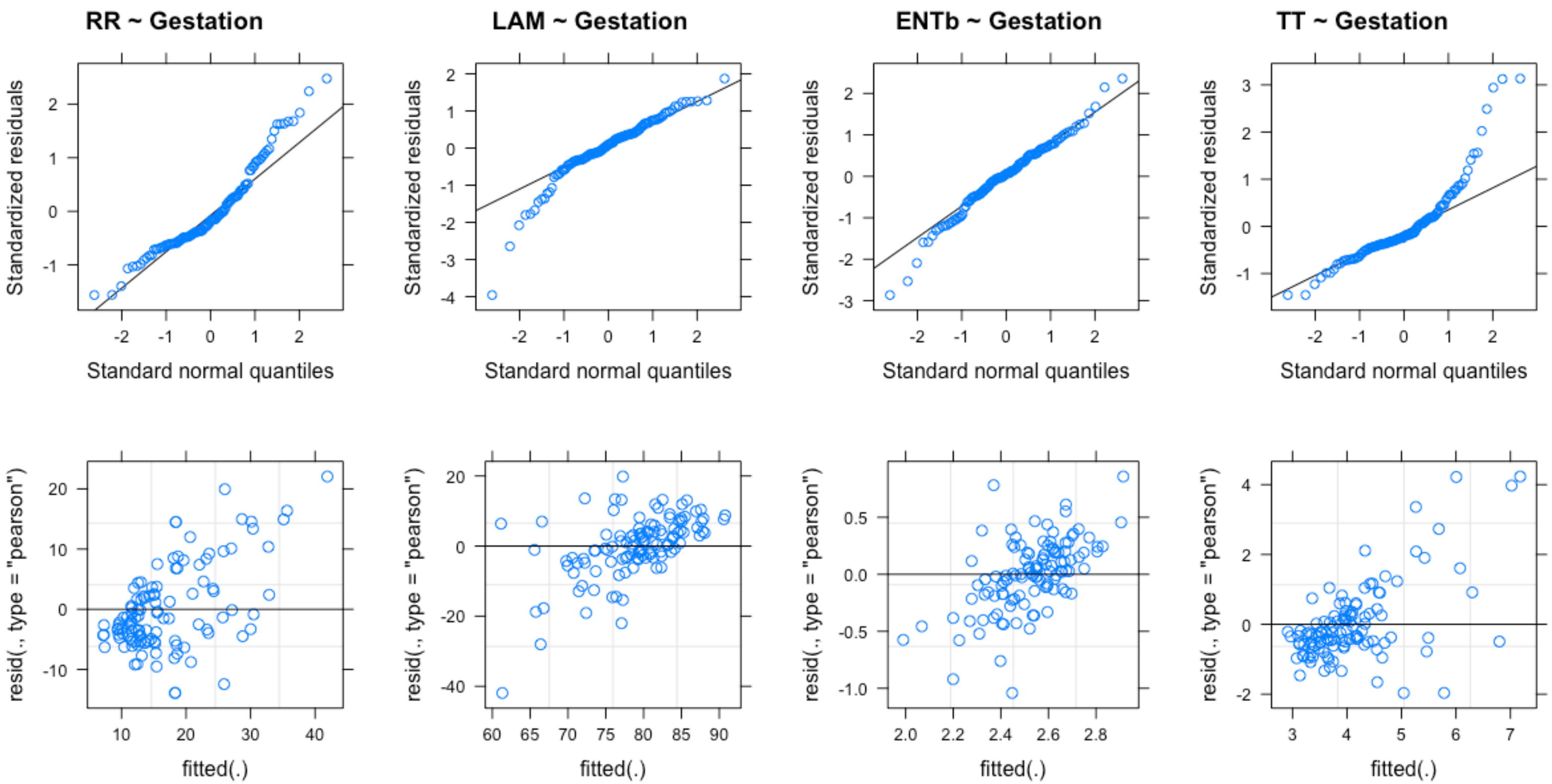

Figure S7. Model B (Term group) diagnostic plots for behavioural dynamics (top: normal q-q plots, bottom: residuals vs fitted)

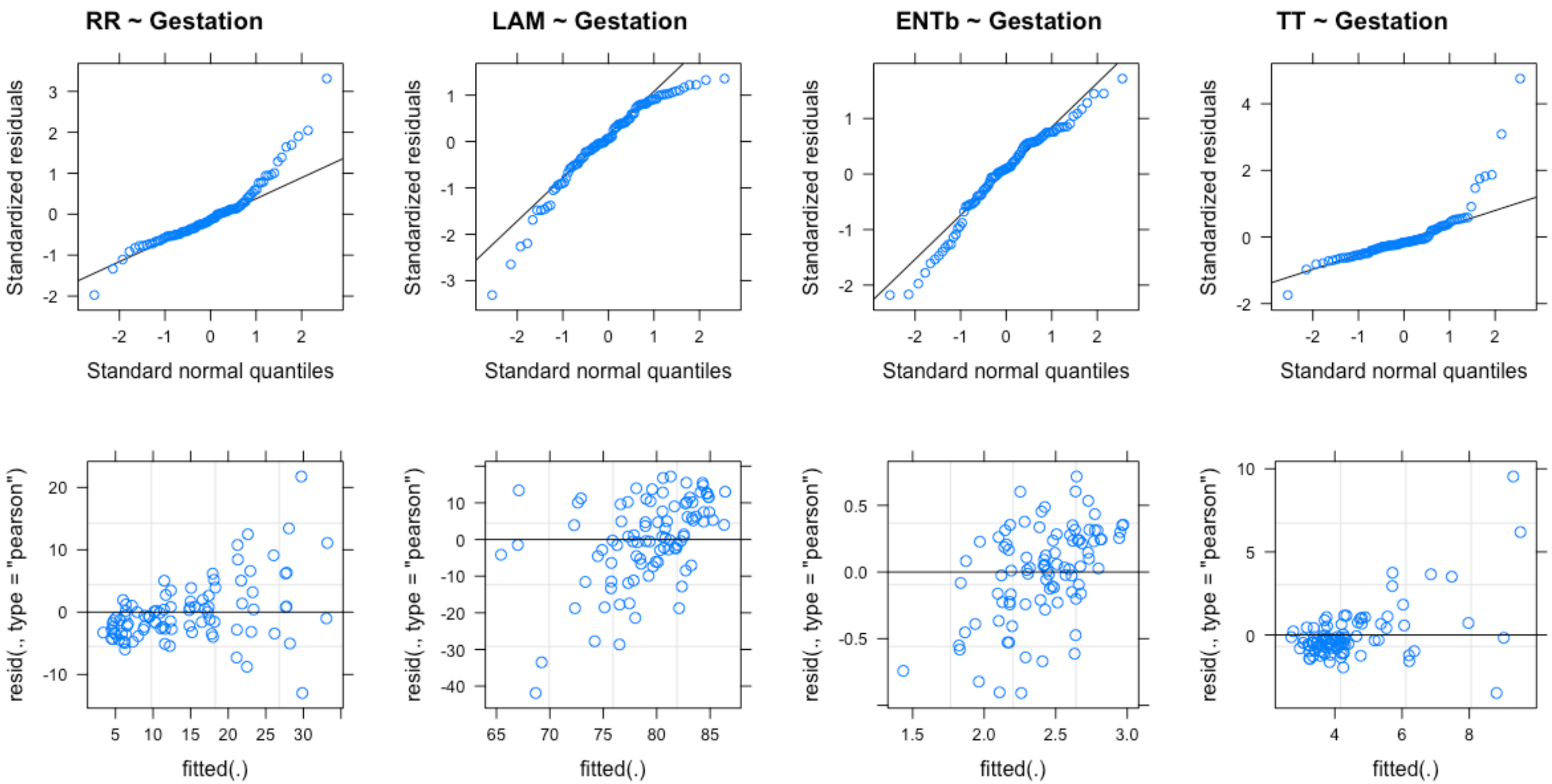

Figure S8. Model B (Preterm group) diagnostic plots for behavioural dynamics (top: normal q-q plots, bottom: residuals vs fitted)

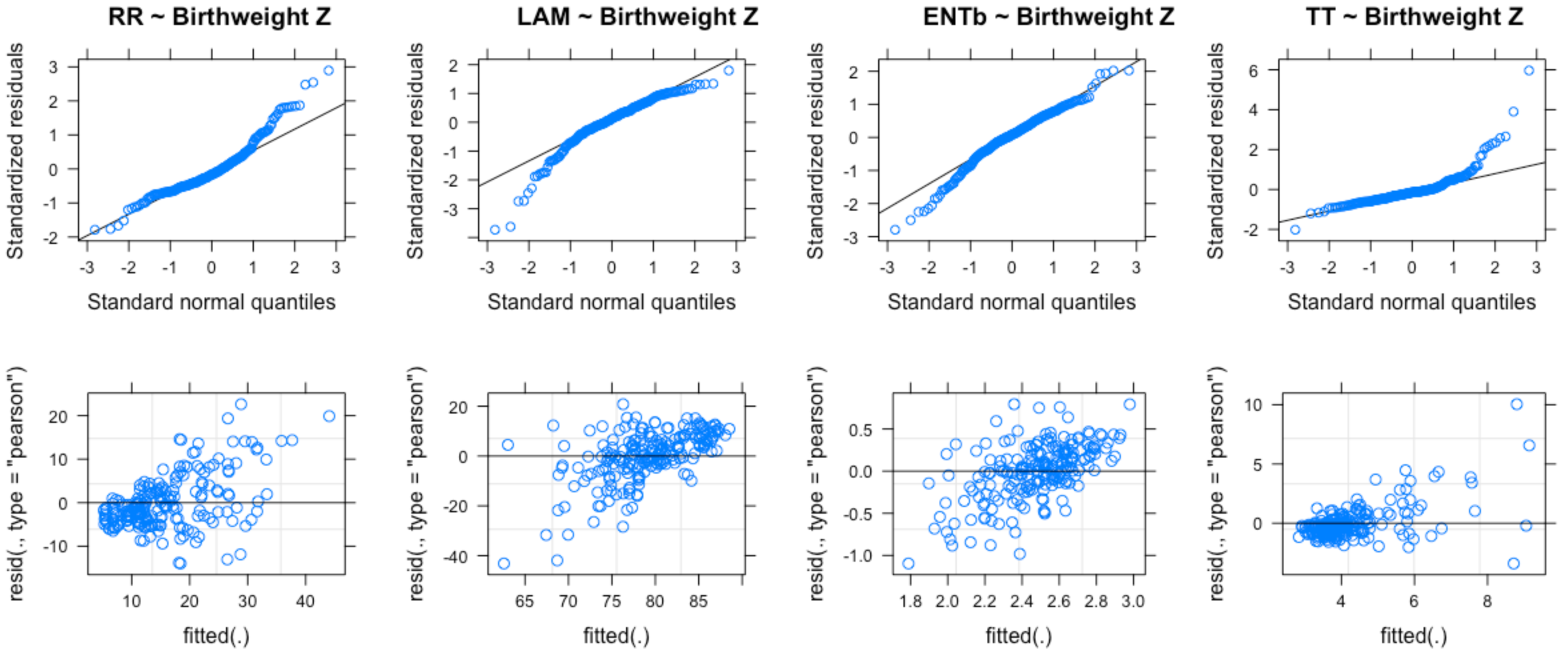

Figure S9. Model C diagnostic plots for behavioural dynamics (top: normal q-q plots, bottom: residuals vs fitted)

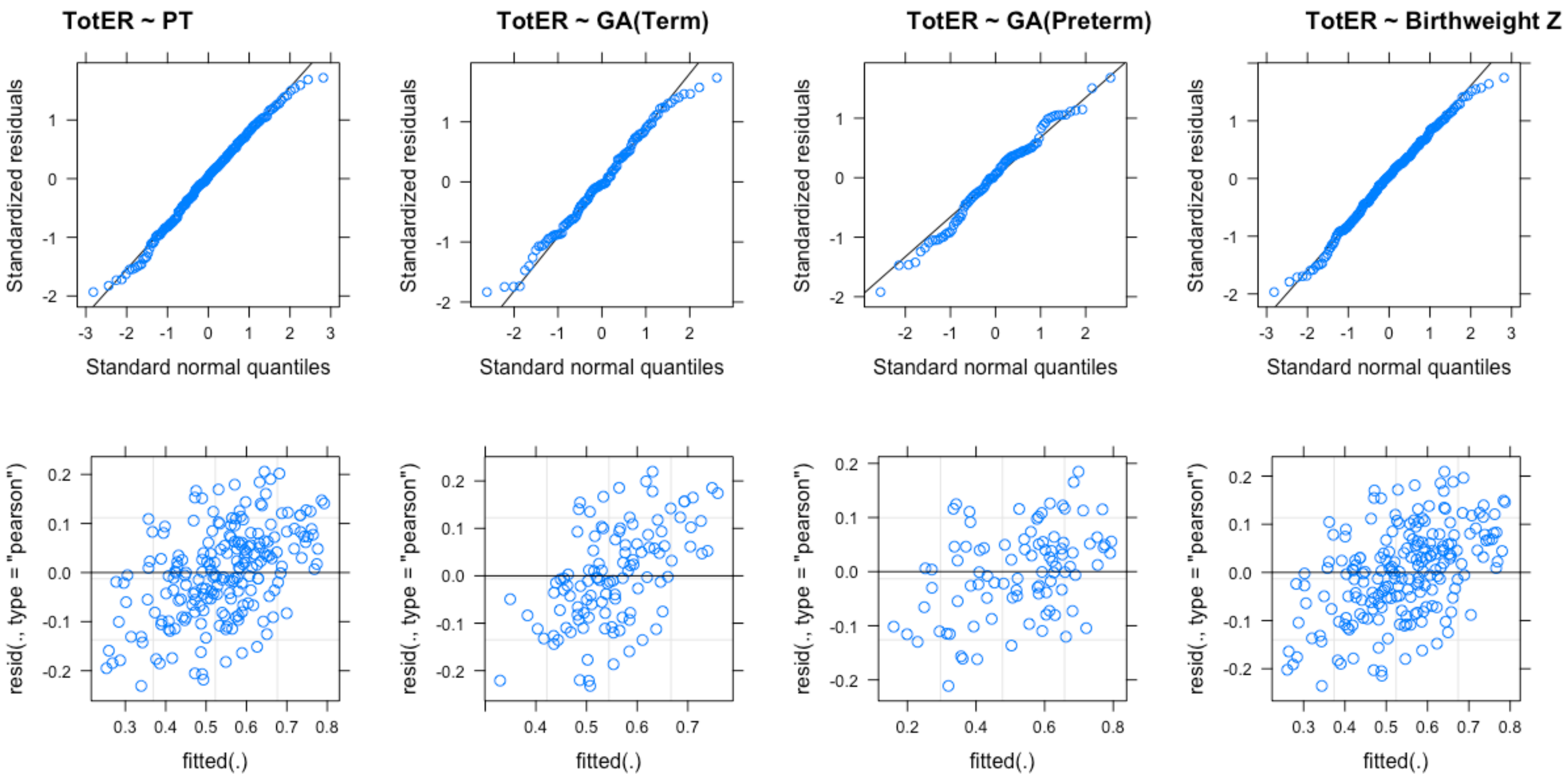

Figure S10. Model D diagnostic plots (top: normal q-q plots, bottom: residuals vs fitted)

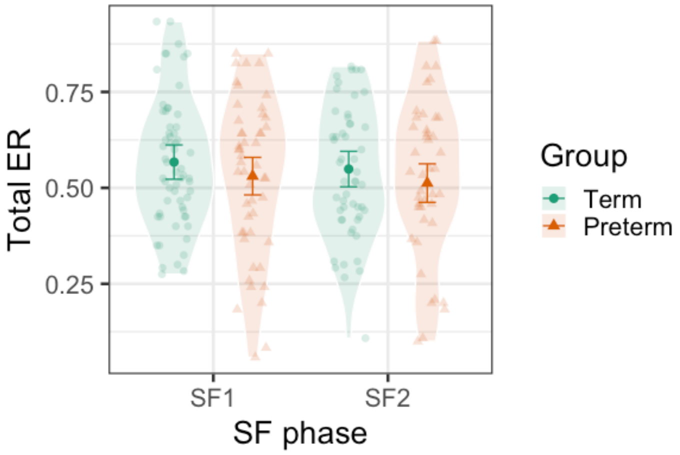

Figure S11. MODELS D – effect of preterm birth on Total ER behaviours. Marginal effects with standard errors overlaid over observed data including violin plot.

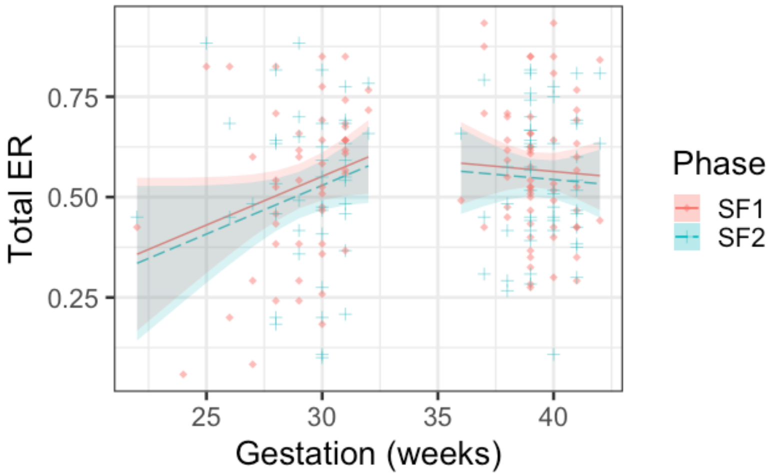

Figure S12. MODELS D – effect of gestational age on Total ER behaviours. Marginal effects with standard errors overlaid over observed data.

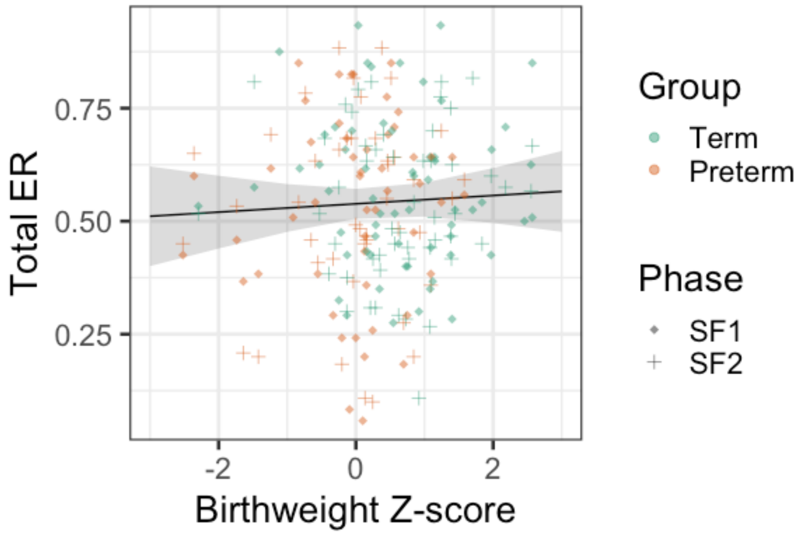

Figure S13. MODELS D – effect of birthweight Z-score on Total ER behaviours. Marginal effects with standard errors overlaid over observed data.

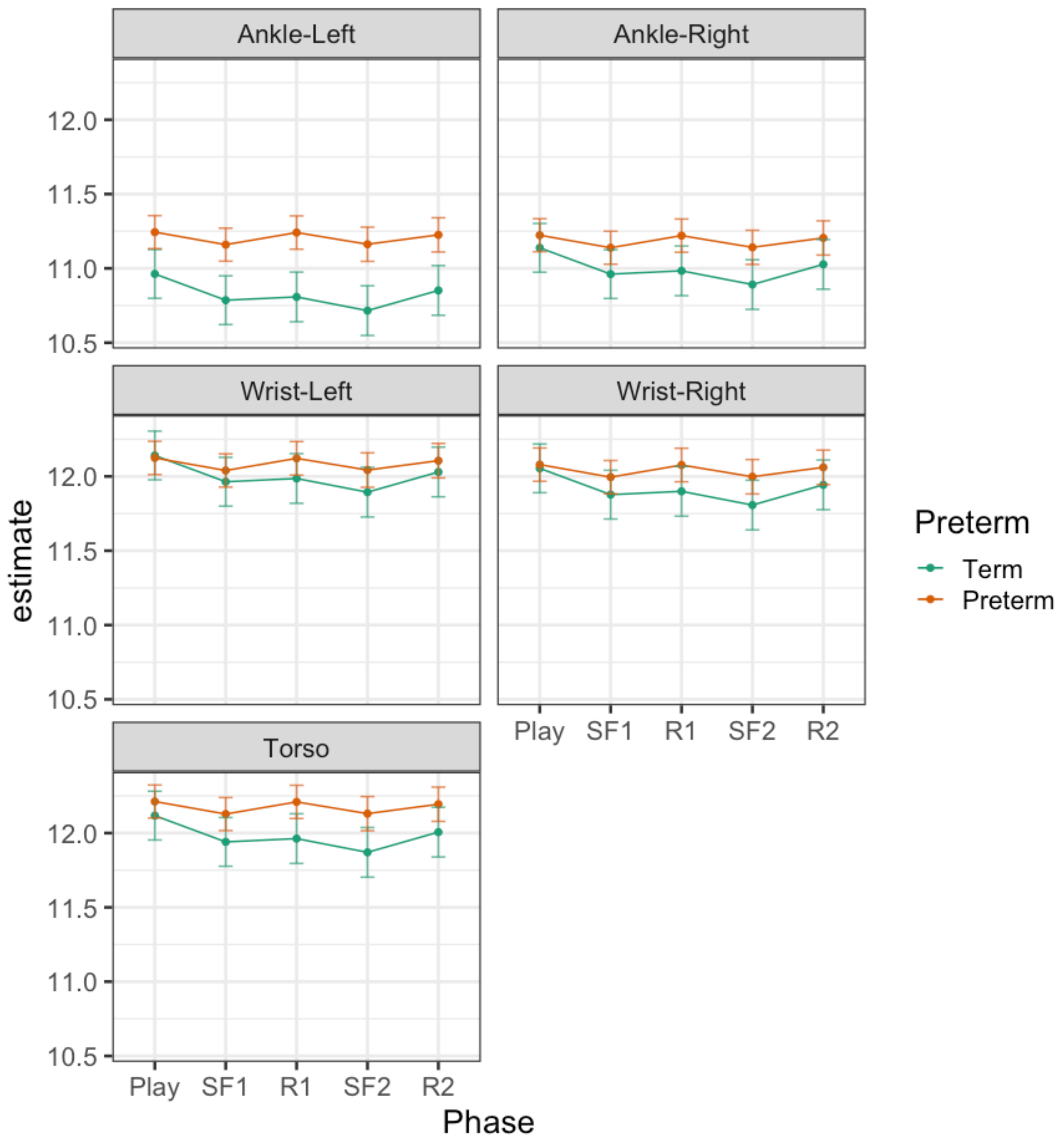

Figure S14. Model results – CI (gamma) – estimated marginal means with confidence intervals

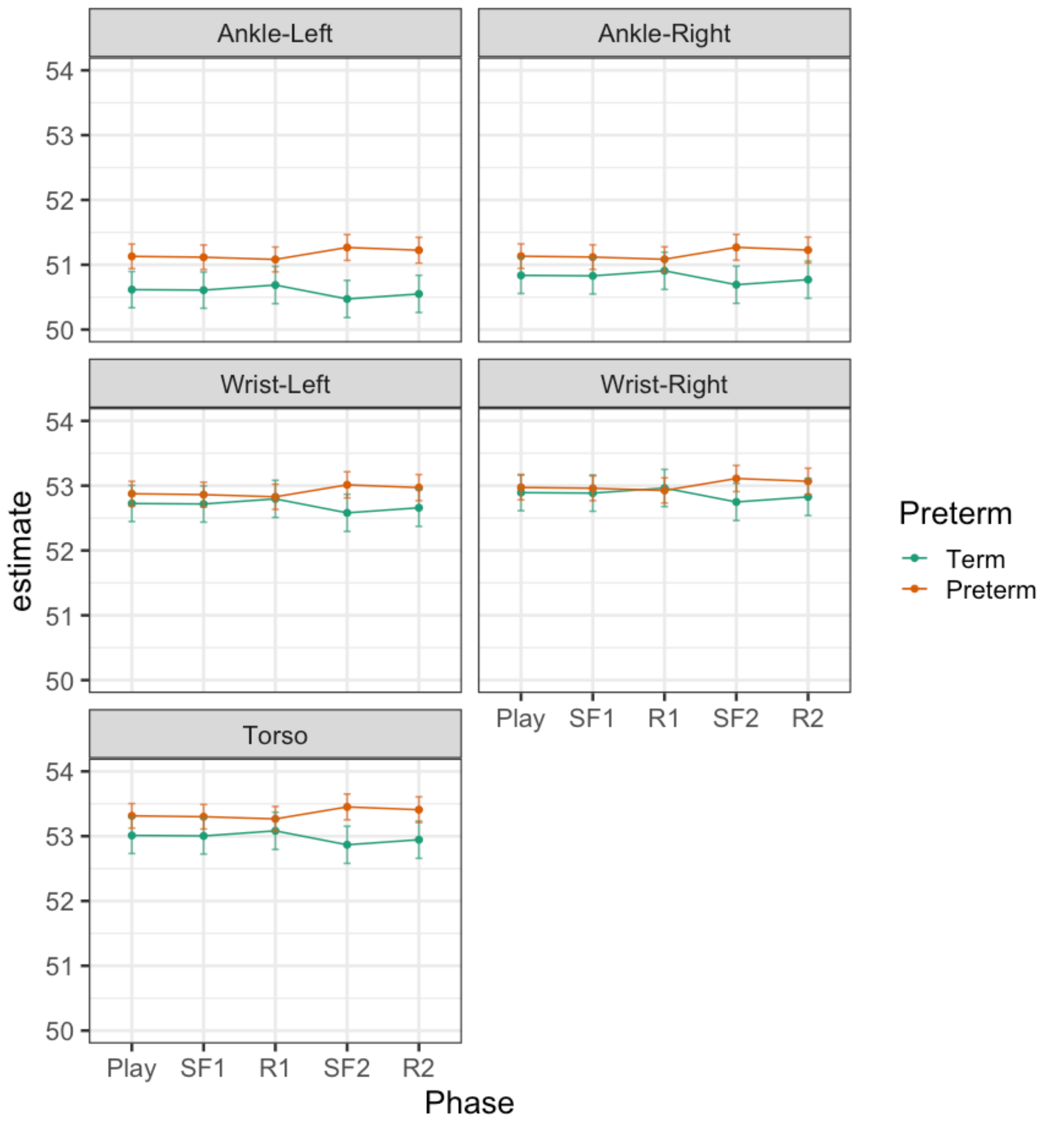

Figure S15. Model results – CI (theta). Estimated marginal means with confidence intervals

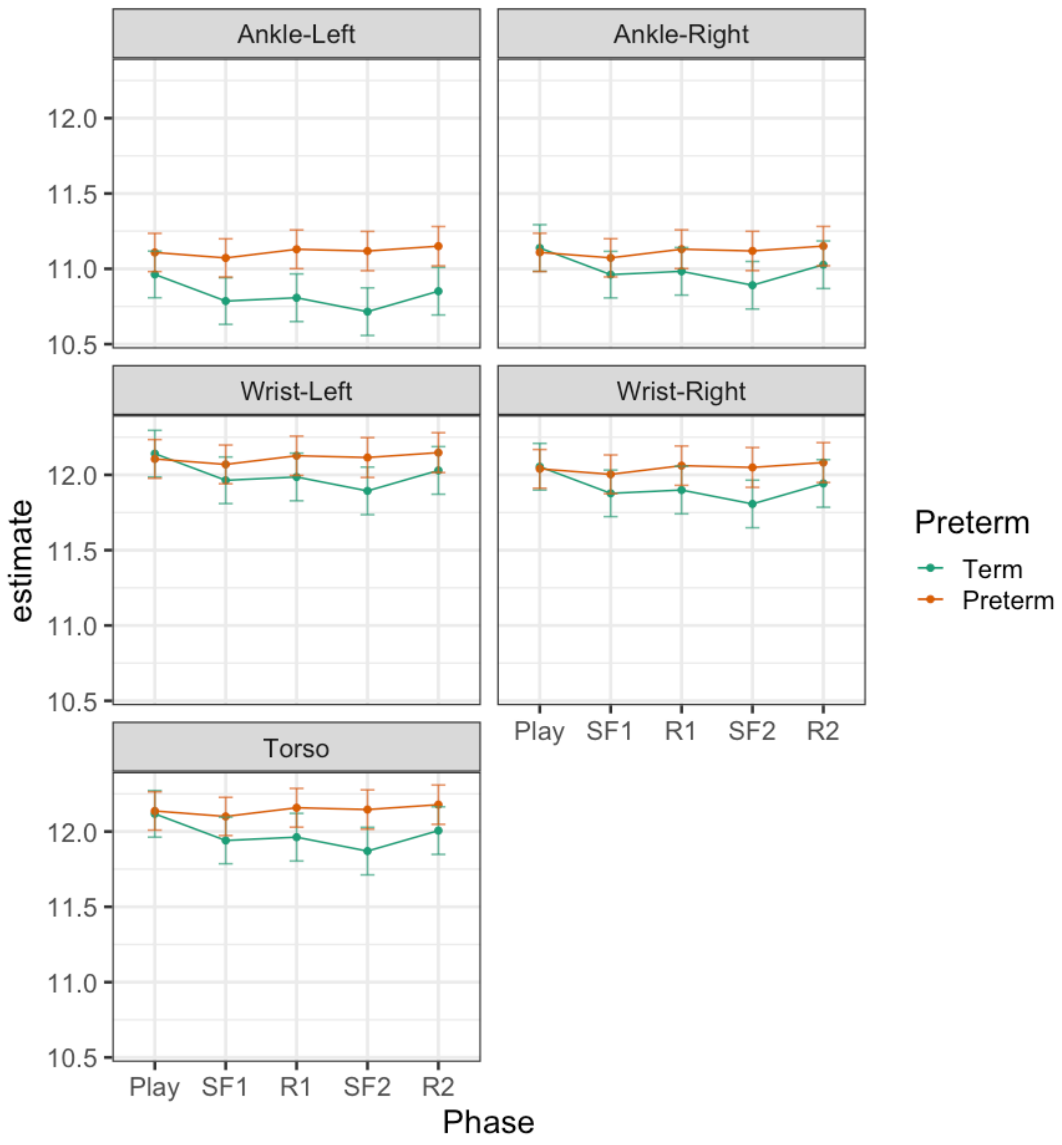

Figure S16. Sensitivity analyses model results – CI (gamma). Estimated marginal means with confidence intervals

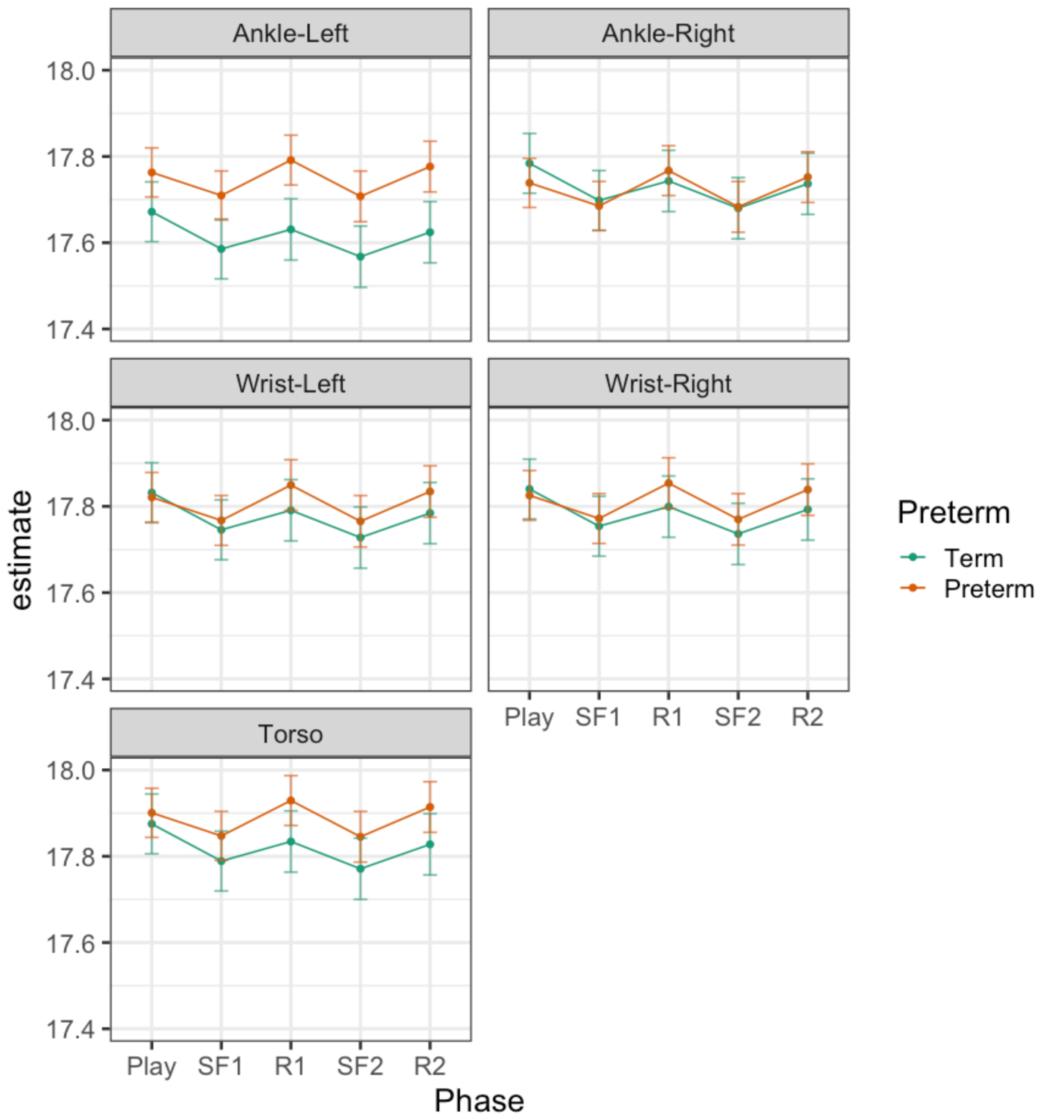

Figure S17. Sensitivity analyses model results – CI (beta). Estimated marginal means with confidence intervals

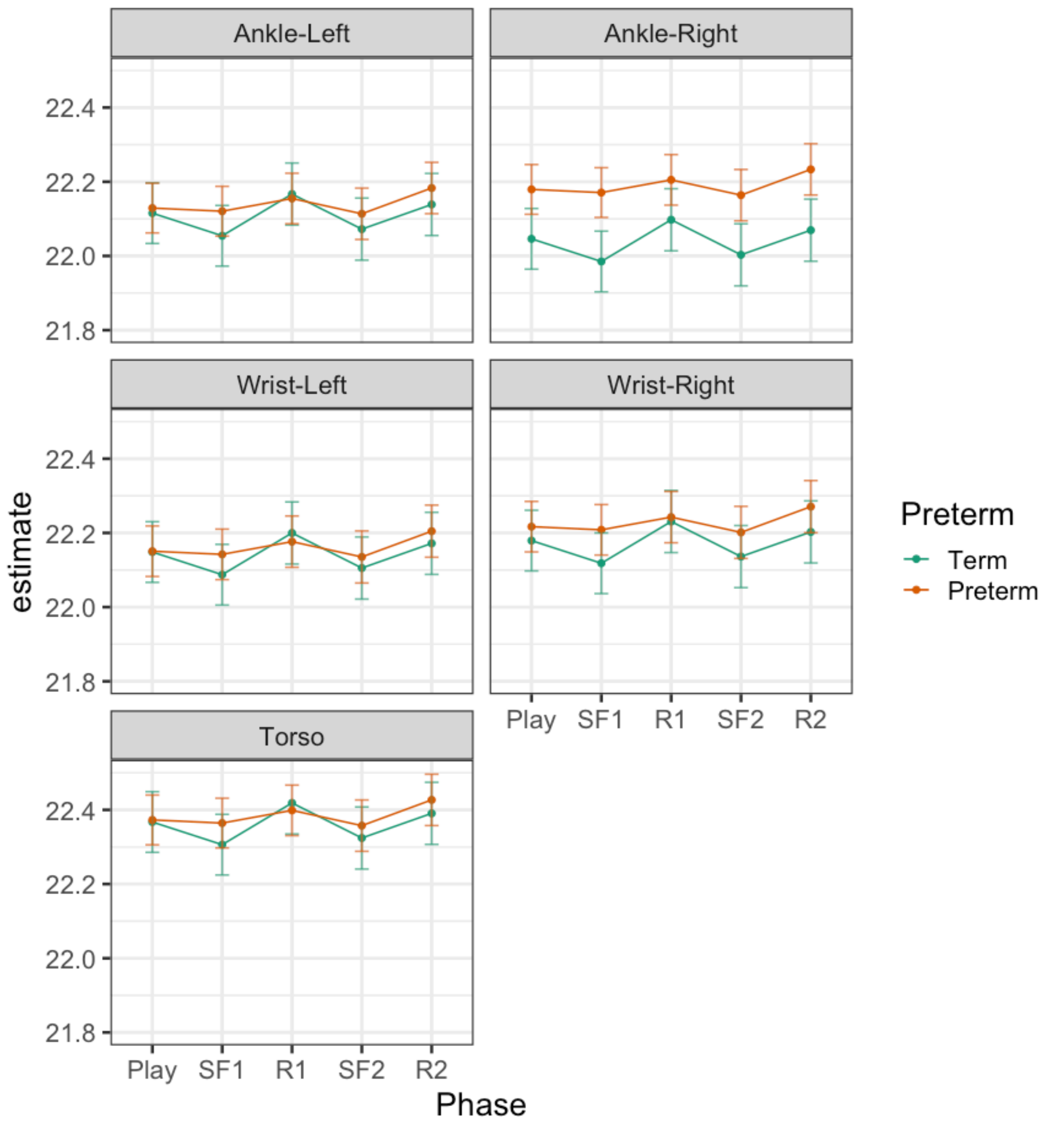

Figure S18. Sensitivity analyses model results – CI (alpha). Estimated marginal means with confidence intervals

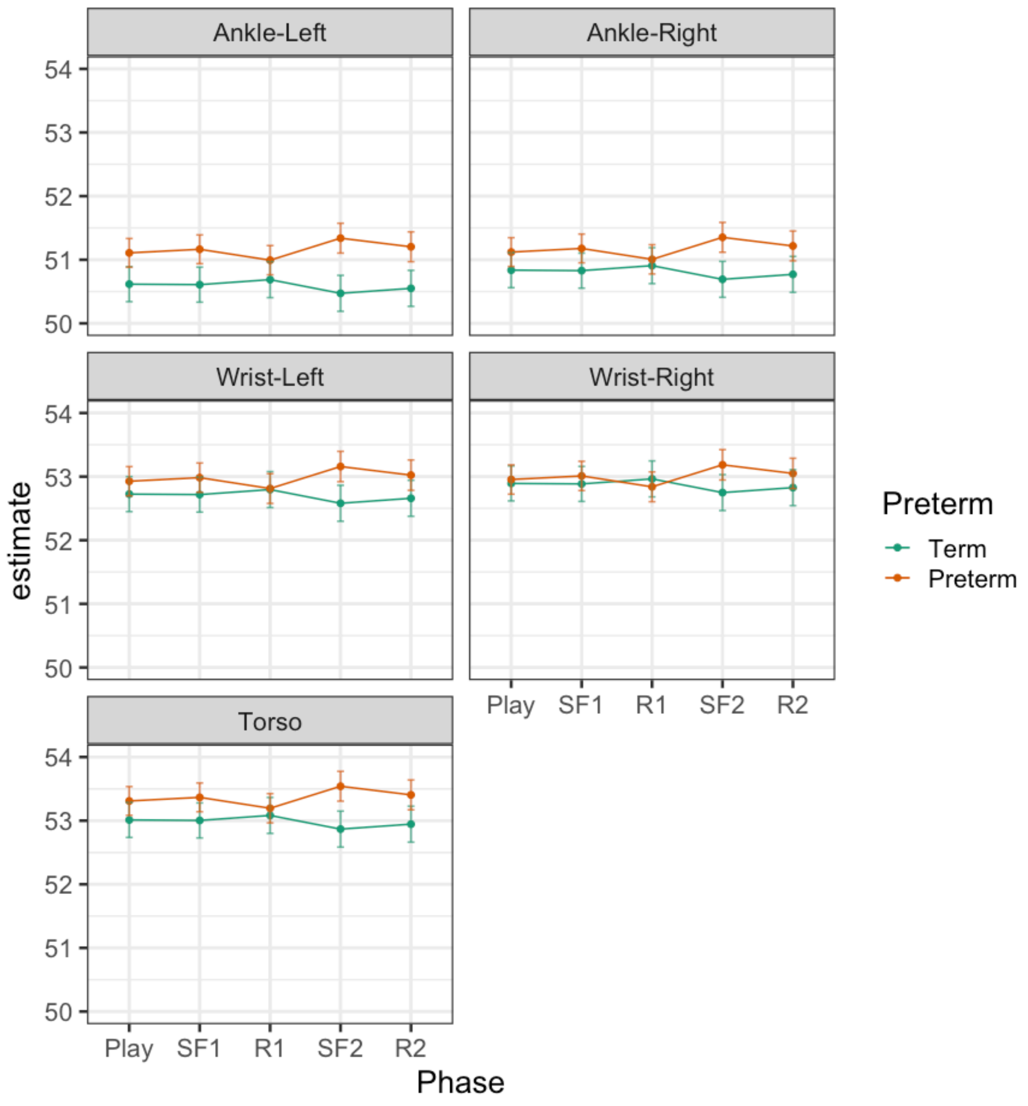

Figure S19. Sensitivity analyses model results – CI (theta). Estimated marginal means with confidence intervals

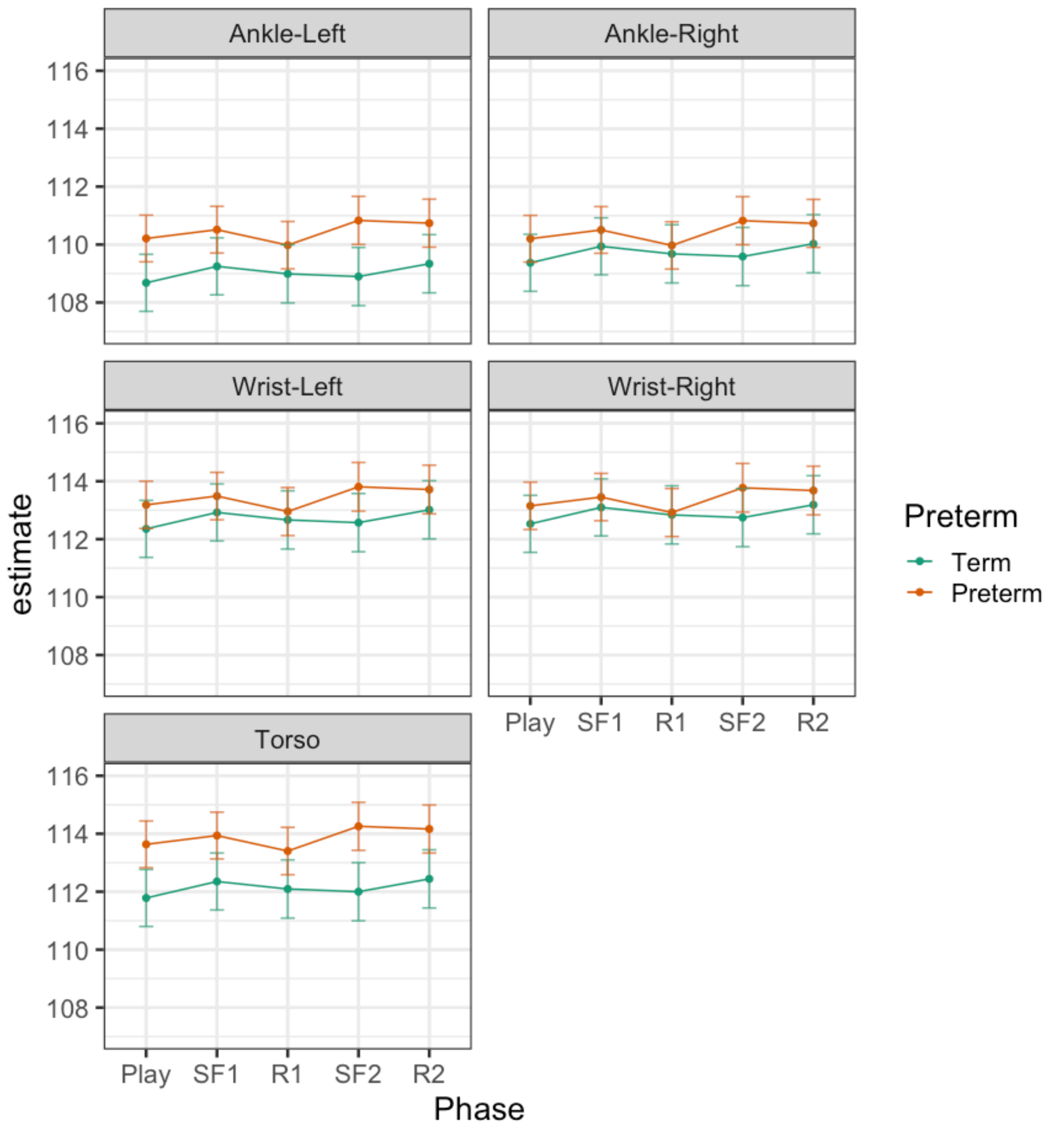

Figure S20. Sensitivity analyses model results – CI (delta). Estimated marginal means with confidence intervals

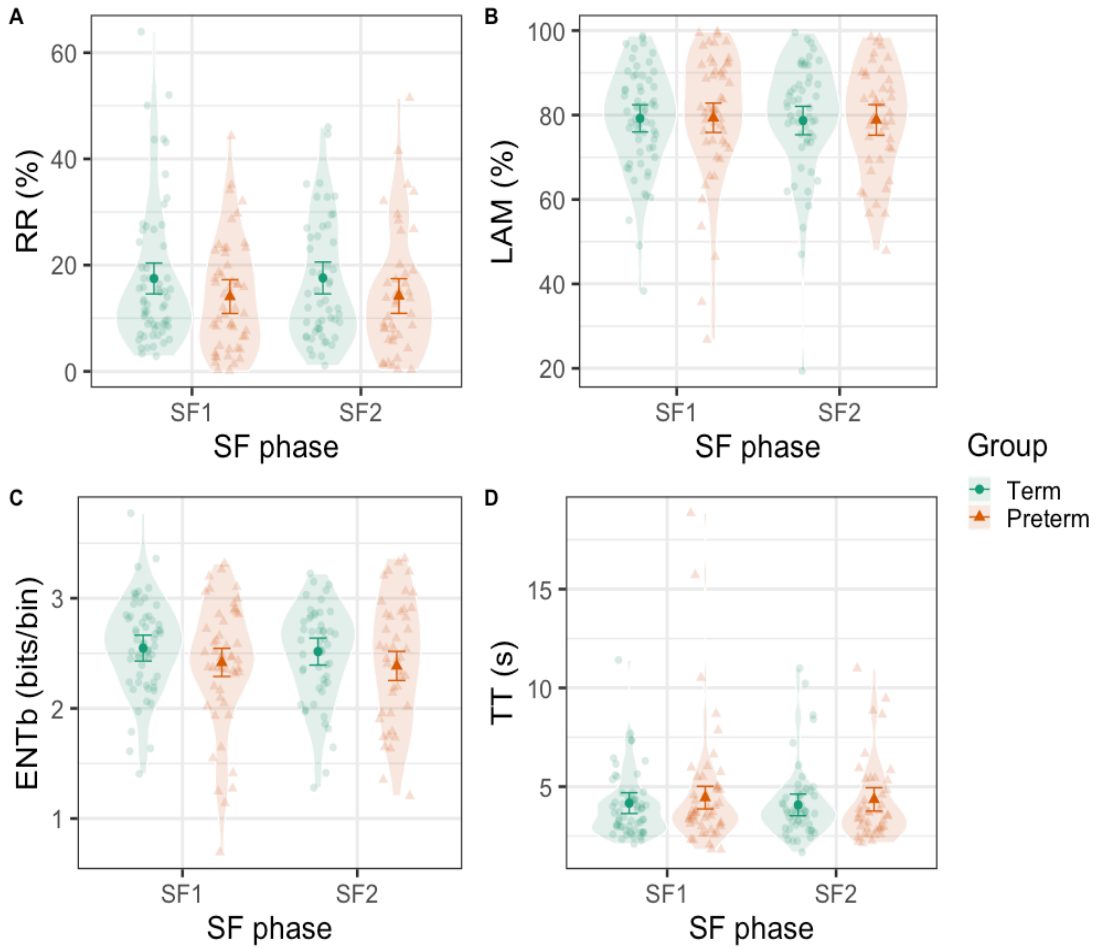

Figure S21. MODELS A – Effect of preterm birth and still-face phase on behavioural dynamics (no interaction effect). Main effects with confidence intervals, overlaid on violin plots and scatterplots of observed data. Panel A: Recurrence Rate (RR); Panel B: Laminarity (LAM); Panel C: Entropy of block structures (ENTb); Panel D: Trapping time (TT)

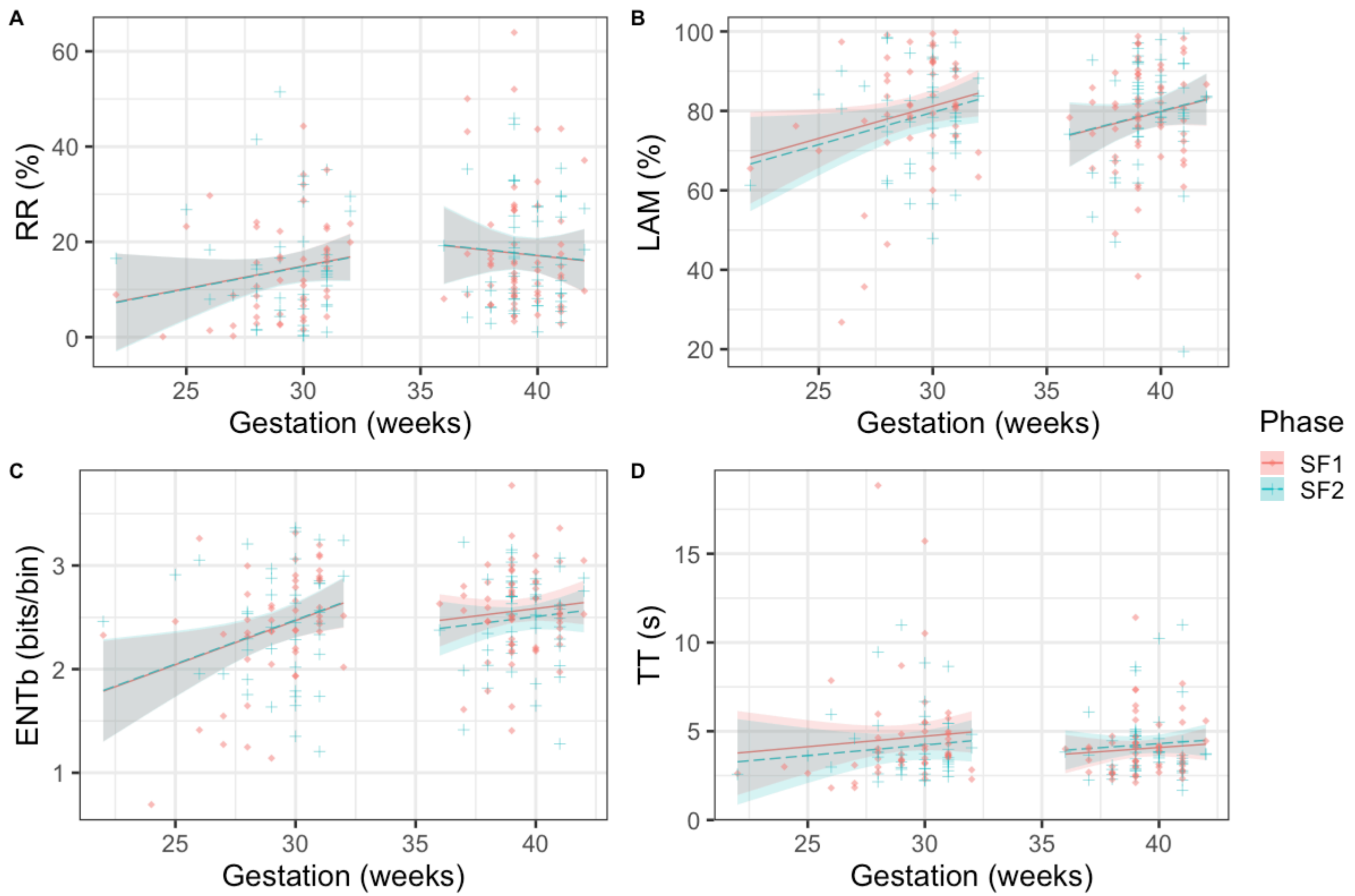

Figure S22. MODELS B – effect of gestational age on behavioural dynamics modelled for preterm and term group separately. Marginal effects with standard errors overlaid on observed data

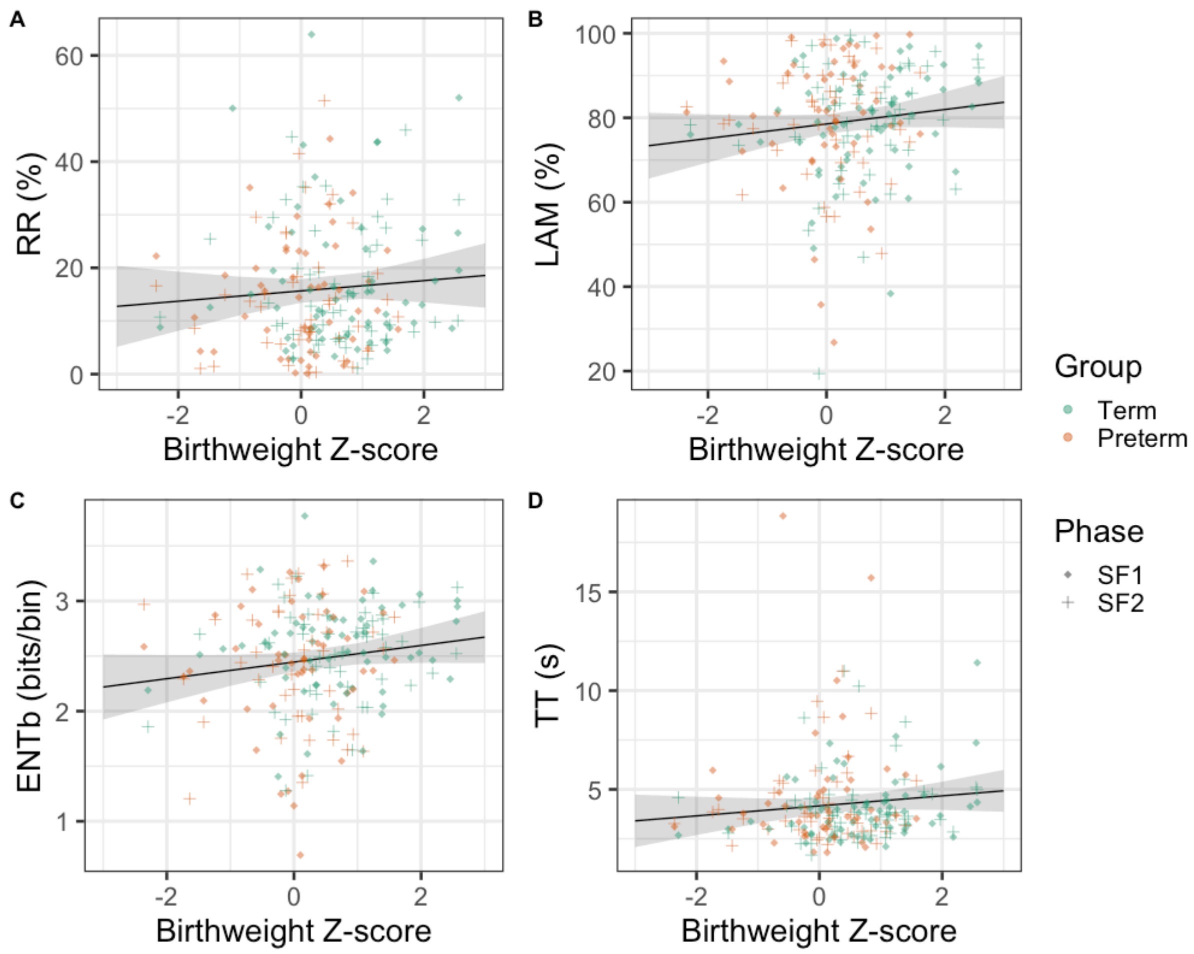

Figure S23. MODELS C – effect of birthweight Z-score on ER behavioural dynamics. Marginal effects with standard errors overlaid over observed data.
